# The chemosensory system of the clonal raider ant larva

**DOI:** 10.64898/2026.10.06.757048

**Published:** 2026-10-09

**Authors:** P. Daniel H. Pastor, Leonora Olivos-Cisneros, Alejandra Hurtado-Giraldo, Anurag Sharma, Patrick K. Piekarski, Giacomo L. Glotzer, Daniel J. C. Kronauer

**Affiliations:** 1 Tri-Institutional MD–PhD Program, Weill Cornell/Rockefeller/Sloan Kettering, New York, NY 10065, USA; 2 Laboratory of Social Evolution and Behavior, The Rockefeller University, New York, NY 10065, USA; 3 Electron Microscopy Resource Center, The Rockefeller University, New York, NY 10065, USA; 4 Howard Hughes Medical Institute, New York, NY 10065, USA

**Keywords:** Chemosensation, Formicidae, Olfaction, Sensory Anatomy, Social Insects

## Abstract

Ant larvae are blind and immobile, depending heavily on the adults for care in the form of feeding, grooming, and transport. However, they are not inactive colony members but play a central role in the organization of ant societies by modulating adult behavior and physiology. While adult ants have been studied extensively, we still know little about what sensory modalities larvae use to perceive their environment. Here, we provide a molecular and circuit-level characterization of the chemosensory system of the clonal raider ant larva. Combining scanning electron microscopy and confocal imaging, we show that dome sensilla in the maxillary and labial palps are innervated by odorant receptor (OR) neurons. A larger population of ionotropic receptor (IR) neurons innervate papilla sensilla across various regions of the larval head: the antennae, the labrum, the maxillary palps and galeae, as well as the labial palps and the ventral labium. This suggests that IRs, which can have various functions, from taste and olfaction to temperature and humidity sensing, constitute the predominant chemosensory modality in ant larvae. To investigate projections into the central brain, we imaged transgenic lines that fluorescently label OR- and IR-expressing neurons. We found that OR- and IR-expressing neurons in the labium and maxilla project along the labial and maxillary nerves through the subesophageal ganglion to the deutocerebrum, whereas IR-expressing neurons from the labrum project to the tritocerebrum and those from the antenna project to the posterior deutocerebrum. Together, our findings establish a structural and molecular framework to study larval chemosensation in ants.

## Introduction

Insect societies are organized around brood care, with adult workers tending to the various immature stages. In ants, larvae are relatively immobile and depend on adults for protection, food, and hygiene. However, larvae are not simply passive recipients of worker care. Increasing evidence shows that larvae actively shape adult behavior and physiology, from foraging behavior to egg-laying (1–10). In many species, they also participate in nutrient circulation via trophallaxis, acting as the colony’s “stomach” (11). In ants and other social insects, chemical communication underlies nearly all aspects of social organization, from non-nestmate discrimination to foraging and brood care (12–14). While the chemosensory systems of adult ants have been studied in considerable detail, the sensory biology of ant larvae remains largely unexplored. Given the prevalence of chemical communication in insect societies and the central role of larvae in colony organization, it is essential to understand how larvae detect and interpret chemical information.

In insects, chemosensation is typically mediated by specialized sensory neurons whose dendrites innervate morphologically distinct types of sensilla. The main chemosensory organs of adult insects are the antennae and mouthparts, where odorant receptors (ORs), ionotropic receptors (IRs), and gustatory receptors (GRs) detect volatile and contact-based chemical cues (15–19). Insect ORs are seven-transmembrane, ligand-gated cation channels consisting of a conserved coreceptor, Orco, and one of many tuning ORs. They detect both volatile and non-volatile odorants. IRs, on the other hand, are ligand-gated ion channels of the ionotropic glutamate receptor superfamily, where a coreceptor (often Ir8a or Ir25a and, more rarely, Ir76b or Ir93a) combines with tuning IRs to form functional receptor complexes (15, 18, 20). IRs have been implicated in contact and volatile chemosensation, predominantly of amines, acids, and other small polar compounds, as well as thermosensation and hygrosensation. Unlike ORs and IRs, GRs, which detect tastants, do not require a conserved coreceptor (21–23).

Insect larvae often lack the complex sensory structures of adults. In *Drosophila melanogaster*, larval chemosensation is mediated by a small number of dedicated sensory organs that project directly to the central brain, reflecting the simpler behavioral needs of larvae (24–27). Classical descriptions of ant larval sensory organs date back over half a century and primarily used light microscopy to examine external anatomy (28–30). Recent μCT studies have also begun to reveal the internal architecture of ant larvae (31). While these studies identified putative sensory structures on the larval head and mouthparts, they provided limited insight into their cellular composition, organization, and function. Moreover, how these peripheral sensory organs connect to the larval nervous system and which chemosensory receptor families they employ remains unresolved.

Here, we investigate the structural and molecular organization of the larval chemosensory system in the clonal raider ant, *Ooceraea biroi*. Using scanning electron microscopy (SEM), we systematically characterize the distribution and morphology of sensilla across the larval head. Using a newly generated *Ir25a* reporter line, together with a previously established *Orco* reporter line, antibody staining, and RNA fluorescent in situ hybridization (RNA-FISH), we identify larval chemosensory neuron populations, define their receptor expression profiles, and trace their projections into the central nervous system. Our results reveal a conserved yet specialized chemosensory architecture, identify dome sensilla as the primary olfactory organs, and demonstrate that ionotropic receptor–expressing cells constitute the largest population of chemosensory neurons in the ant larva. Together, these findings provide a foundation for understanding how larval chemosensation operates in the context of development and ant social life.

## Results

### An Inventory of Sensory Organs and Sensilla on the Larval Head

George and Jeanette Wheeler described the external morphology of ant larvae across species, including the number of sensilla on the antennae, labrum, maxilla and labium (28, 29). However, their studies mostly relied on light microscopy, and the fine structure of these sensilla has therefore not been investigated in detail. We initially imaged larvae across all four instars using SEM. Because the same sensory structures were present across all instars, subsequent analyses focused on the fourth and final instar, which is the largest and therefore provides the clearest structural resolution in both SEM and confocal imaging. Examples of sensory structures of early-instar larvae are shown in Figure S1 for comparison.

The major regions of the larval head where chemosensory sensilla occur are the frons, labrum, paired maxillae with palps and galeae, mandibles, and the labium (Fig. 1A,B). In these regions, we identified three major sensillum types: papilla sensilla, rod sensilla, and dome sensilla (Fig. 1C–E). At the top of the head, within the frons, we identified six papilla sensilla arranged in two bilateral clusters (Fig. 1F). These clusters correspond to the structure historically referred to as the larval “antenna” (28, 29). Four additional papilla sensilla occur on each side of the labrum, with three positioned along the external surface and a fourth located more medially and internally (Fig. 1G).

Both the maxillary and labial palps contain multiple sensilla types. The maxillary palps exhibit a stereotyped, bilaterally symmetric organization, with each side containing two papilla sensilla, two dome sensilla with surface perforations, and a single rod-shaped sensillum positioned medially (Fig. 1H). The galeae project anteriorly from the maxillae and each contains two papilla sensilla at the tip (Fig. 1I). The labial palps display a similar organization as the maxillary palps, with two dome sensilla and one rod-shaped sensillum on each side, but one instead of two papilla sensilla compared to the maxillary palps (Fig. 1J). In addition, a single papilla sensillum is consistently present on each side at the ventral base of the labium (Fig. 1K). The distribution, types, and number of sensilla identified across the larval head are summarized in Table 1.

### Dome Sensilla Form the Main Olfactory Organ

To identify odorant receptor neurons, we stained whole larvae with an anti-Orco antibody and imaged them with a confocal microscope. Orco-positive cell bodies are distributed bilaterally across the mouthparts in both the maxilla (approximately 11 cells on each side; n = 3 biological replicates) and the labium (approximately 9 cells on each side; n = 3) (Fig. 2A, Table S1). In both regions, dendrites from these neurons project exclusively to dome sensilla, located on the maxillary and labial palps, respectively (Fig. 2B, C). SEM revealed that the dome sensilla on both labial and maxillary palps are multiporous (Fig. 1E). This morphology is consistent with an olfactory function and resembles the well-characterized dorsal organ, the olfactory organ of *D. melanogaster* larvae, which forms a large dome-shaped surface with multiple pores (27, 32–36).

To identify candidate tuning ORs expressed in these Orco-positive neurons, we analyzed bulk RNA-sequencing (RNA-seq) data from heads of fourth-instar *O. biroi* larvae (Fig. S2). We ranked ORs by their expression level and designed RNA-FISH probes for 24 out of the 50 most highly expressed receptors. For unknown reasons, RNA-FISH failed to verify expression of most of these ORs (Fig. S2). Ultimately, we were able to spatially localize the expression of only four ORs identified by RNA-seq using RNA-FISH. One receptor, *OrW1*, is consistently expressed in one Orco-positive cell in each maxilla (n=3) (Fig. 2D–G). The second receptor, *OrE3*, is consistently expressed in one Orco-positive cell on each side of the labium (n=3) (Fig. 2H–K). Two additional ORs show non-canonical expression. *OrL3* is expressed in Orco-negative cells in the larval head and *Or9E137* is expressed in both Orco-positive and Orco-negative cells in the mouthparts. The tuning ORs expressed in the remaining Orco-positive neurons remain unknown. Interestingly, the vast majority of the OR genes in the *O. biroi* genome are arranged in genomic tandem arrays (~469 out of ~500) and their expression in adults is regulated by an unconventional mechanism of transcriptional interference (37–39). The two ORs for which we verified larval expression exclusively in Orco-positive cells are among the ~31 singleton OR genes in the genome (39). This opens the possibility that *O. biroi* larvae, unlike the adults, primarily express singleton ORs.

### IRs are Broadly Expressed in Chemosensory Cells of the Larval Head

To examine the expression of IRs in the larval head, we conducted RNA-FISH for the four canonical IR coreceptors (Figs. S3 and S4). Three of the four coreceptors—*Ir25a*, *Ir8a*, and *Ir76b*—were detected in sensory cells, whereas no *Ir93a* expression was detected by RNA-FISH. All three detected coreceptors were expressed across the antennae, dorsal esophageal region, labrum, maxillae, and labium, although their relative abundance varied among regions (Table S1). *Ir25a* and *Ir8a* showed the broadest expression, with *Ir25a* detected in ~239 cells across the larval head (n = 3) and *Ir8a* in ~232 cells (n = 1), whereas *Ir76b* was detected in ~96 cells (n = 3) (Figs. S3 and S4; Table S1). Nearly all *Ir8a*-positive cells also expressed *Ir25a* (97% across the whole head), with 94–100% co-expression across individual anatomical regions (n = 1; Fig. S3; Table S2). Most *Ir76b*-positive cells likewise expressed *Ir25a* (87% across the whole head; n = 3), although the relative intensities of the coreceptor signals varied among colabeled cells (Fig. S3; Table S2). Given the broad expression of *Ir25a* and its extensive overlap with the other IR coreceptors, as well as its well-characterized role as an IR coreceptor in other insects (15, 18, 20, 40–65), we focused subsequent analyses on *Ir25a*.

*Ir25a* is expressed across multiple regions of the larval head, with prominent labeling in sensory organ regions of the labium (~52 cells), maxilla (~82) and labrum (~64) (Table S1). Additional *Ir25a*-positive cells are found in two internal populations: ~18 cells between the labrum and brain near the esophagus, hereafter referred to as the dorsal esophageal population, and ~22 cells dorsal to the brain near the antennae, hereafter referred to as the antennal population (n = 3 for all counts) (Table S1).

RNA-FISH co-staining for *Ir25a* and *Orco* in the larval head showed that both coreceptors are expressed in the labial and maxillary regions, adjacent to each other but in largely separate cell populations (Fig. S5). Weak *Ir25a* signal was detected in some *Orco*-positive cells, but this signal was substantially reduced compared to unambiguously *Ir25a*-positive cells.

To determine which peripheral sensory structures the *Ir25a*-expressing neurons innervate, we generated a new transgenic reporter line expressing the genetically encoded calcium indicator GCaMP7s under control of an *Ir25a* promoter fragment (Fig. S6). This line labels cell bodies, dendrites, and axons, and recapitulates approximately 40% of the endogenous Ir25a-positive cell population, as determined by RNA-FISH (~95 out of ~239 cells; n=3) (Fig. S7; Table S3). GCaMP expression was detected in subsets of *Ir25a*-positive cells across the larval head. On average, GCaMP was detected in 13/22 *Ir25a*-positive antennal cells (~59%), 30/64 labral cells (~47%), 35/82 maxillary cells (~42%), 15/52 labial cells (~30%), and 2/18 dorsal esophageal cells (~11%) (n = 3; Fig. S7; Table S3). Conversely, all GCaMP-positive cells were also *Ir25a* positive (100% concordance across regions; Fig. S7; Table S3). Although the GCaMP reporter did not perfectly recapitulate endogenous *Ir25a* expression, it labeled the major anatomical regions associated with *Ir25a*-positive neurons. Thus, the reporter provides a broad, although incomplete, representation of the *Ir25a*-expressing sensory system.

Figure 3A provides an overview of GCaMP expression across the larval head in this new transgenic line. We next used complementary two-photon and confocal imaging to identify the sensilla innervated by GCaMP/*Ir25a*-positive neurons. Approximately 13 GCaMP/*Ir25a*-positive neurons are located dorsal to the brain and innervate the six papilla sensilla located on the frons that make up the larval antennae (Fig. 3B; Table S3). This provides the first direct demonstration that these structures are innervated by chemosensory neurons, supporting their proposed sensory function based on earlier anatomical descriptions (28, 29). In the labrum, ~30 GCaMP/*Ir25a*-positive neurons innervate the 8 papilla sensilla arranged from medial to lateral and oriented anteriorly (Fig. 3C; Table S3). The dorsal esophageal region contains 2 GCaMP/*Ir25a*-positive neurons (Table S3). The projections of these cells were difficult to trace because their dendrites disappear upon exiting the soma and presumably innervate the nearby esophagus. In the maxillae, ~35 GCaMP/*Ir25a*-positive neurons innervate papilla sensilla on both the galeae and maxillary palps (Fig. 3D–F; Table S3). Fifteen GCaMP/*Ir25a*-positive neurons innervate the single papilla sensillum on each labial palp and the paired sensilla at the base of the labium (Fig. 3G and H; Table S3).

To determine which IR tuning receptors are expressed in *Ir25a*-positive chemosensory neurons, we ranked candidate receptors based on their expression levels in bulk RNA-seq data from larval heads and performed RNA-FISH on 10 of the 22 annotated tuning IRs (66), spanning the entire range of expression levels (Fig. S2). Unlike for tuning ORs, the RNA-FISH signal intensities and cell numbers for tuning IRs generally matched expectations from the transcriptomic data (Fig. S2; Table S1). The two tuning IRs with the highest transcript abundance based on RNA-seq, *Ir317.2* and *Ir75f.3*, were detected by RNA-FISH in 24 and 11 *Ir25a*-positive cells, respectively (n = 3; Fig. 3I–M; Fig. S8; Table S1). *Ir317.2* was detected in all anatomical regions except the dorsal esophagus, whereas *Ir75f.3* was detected in the labrum, maxillae, and labium but not in the antennae or dorsal esophagus. *Ir308*, which ranked third in transcript abundance, showed the broadest cellular distribution, with expression detected in 77 cells distributed across all anatomical regions (n = 3; Fig. S9; Table S1). By contrast, *Ir75u.1*, which had low RNA-seq transcript abundance (Fig. S2), was restricted to two cells, one in each maxilla (n = 3; Fig. S9; Table S1). The remaining six tuning IRs tested by RNA-FISH, all of which had low RNA-seq transcript abundance, were not detected (Fig. S2).

### GRs are Expressed in Similar Regions of the Larval Head as IRs

GRs constitute the third major class of insect chemoreceptors, and we asked whether they are expressed in the same regions of the larval head as ORs and IRs. We ranked candidate GRs by transcript abundance in bulk RNA-seq data from larval heads (Fig. S2) and performed RNA-FISH for the four most highly expressed GRs. These four GRs showed broad but distinct distributions across the larval head (n = 3; Figs. S10 and S11; Table S1). *Gr25*, the most highly expressed GR according to RNA-seq, was detected in the antennae (~6 cells), dorsal esophageal region (~4), labrum (~27), maxillae (~19), and labium (~15), for a total of ~71 cells (Table S1). Co-staining further showed that *Gr25* frequently overlaps with the IR coreceptor *Ir25a*: 61% of *Gr25*-positive cells across the whole head also expressed *Ir25a*, with regional overlap ranging from 39% in the maxillae to 100% in the antennae (n = 1; Table S2). Only 5% of *Gr25*-positive cells were positive for both *Ir25a* and *Orco* (n = 1; Table S2). *Gr6* was similarly widespread, with ~8 cells in the antennae, ~4 in the dorsal esophageal region, ~40 in the labrum, ~21 in the maxillae, and ~16 in the labium (~89 cells total) (Fig. S10). *Gr8* was detected in ~2 antennal, ~25 labral, ~39 maxillary, and ~30 labial cells, with no detectable expression in the dorsal esophageal region (~96 cells total) (Table S1). *Gr13* was detected in all five regions, with ~11 antennal, ~5 dorsal esophageal, ~20 labral, ~8 maxillary, and ~10 labial cells (~54 cells total) (Fig. S11; Table S1).

### IR- and OR-expressing Neurons Project to the Brain Via Both Private and Shared Axonal Tracts

So far, we have shown that, in *O. biroi* larvae, OR neurons innervate dome sensilla on the maxillary and labial palps, IR neurons innervate papilla sensilla on the antennae, labrum, maxilla, and labium, and GR neurons occupy the antennal, labral, maxillary, and labial regions. We next examined the axonal projections of these distinct populations of chemosensory neurons to determine whether they converge onto a single neuropil in the central brain, or whether they project in a more distributed manner. Because the ant larval brain has not been described or annotated, we relied on the terminology established in honeybee (*Apis mellifera*) larvae and general insect neuroanatomy (67, 68). This included defining the deutocerebrum and tritocerebrum of the central brain (Fig. 4A and B). In insects, the deutocerebrum is the primary brain region associated with olfactory input, including antennal nerve projections when antennae are present (67), while the tritocerebrum receives innervation from the labrum (69, 70).

Unlike in adults, Orco immunostaining in larvae did not label axonal tracts, suggesting that in larval olfactory sensory neurons the Orco protein is not substantially trafficked into axons (Fig. S12M–P). This also means that Orco immunostaining is insufficient to track axonal projections into the central brain. We therefore imaged larval Orco-expressing neurons and their processes in a previously generated transgenic line that expresses GCaMP6s in OSNs under an *Orco* promoter fragment (71). Because this line has so far only been characterized in adults (71, 72), we assessed labeling specificity in larvae by co-staining with an anti-Orco antibody. In the maxillae and labium, where Orco-positive neurons are located, most GCaMP-positive neurons were also Orco-positive, indicating strong correspondence between reporter labeling and endogenous *Orco* expression (Fig. S12 and Table S3). One GCaMP-positive neuron in the maxilla lacked detectable Orco signal, while another had faint immunostaining, consistent with off-target or leaky reporter expression in Orco-negative neurons (Fig. S12C). We observed additional off-target expression in the labrum, which lacks Orco-expressing cells (Fig. S12O). Finally, one labial neuron was Orco-positive but GCaMP-negative, whereas the corresponding contralateral labial neuron was Orco-positive and showed faint GCaMP signal (Fig. S12F). GCaMP-positive cells in the maxillae and labium, where they are predominantly Orco-positive, project to the subesophageal ganglion (SEG) through the maxillary and labial nerves, respectively. These projections enter the SEG, appear to partially synapse there, and then continue through the circumesophageal connective (CEC) to the deutocerebral region of the central brain, without crossing over (Fig. S12L and M).

To trace the projections of IR-positive neurons in the maxillae and labium and to compare them to those of Orco-positive neurons, we examined our new *Ir25a*-GCaMP line. Broadly, the trajectories of the maxillary and labial projections of *Ir25a*-positive neurons follow those seen in the *Orco*-GCaMP line. These projections travel ventrally into the SEG, where they form multiple presumptive synaptic sites, before continuing dorsally into the deutocerebrum through the CEC (Fig. 4C and D).

In contrast, *Ir25a*-positive labral neurons project to a distinct, more dorsal and anterior brain region, the tritocerebrum (Fig. 4E). Labeling entering the tritocerebrum appears to partially cross the midline through the tritocerebral commissure (Fig. 4F). The antennal neuronal population associated with the three papilla sensilla appears to project to a region posterior to the deutocerebrum signal from the SEG (Fig. S13B–D). Projections from the SEG, labrum, and antennal region form a semi-continuous field of punctate labeling in the central brain (Fig. S13C).

## Discussion

Despite extensive investigation of chemosensation in adult ants, the larval chemosensory system of ants has remained largely unexplored. Previous studies have described the external sensory structures of ant larvae using light microscopy, without elucidating their fine structures, molecular receptor compositions, dendritic innervations, and central neural projections (28–30, 73). Here, we characterize the *O. biroi* larval chemosensory system across these levels, linking external sensilla to receptor-defined neuronal populations and following these populations into the central nervous system. Our results reveal a small and stereotyped set of peripheral sensilla that supports considerable molecular diversity and multiple anatomically distinct central pathways.

SEM identified three major classes of sensilla (papilla, dome, and rod sensilla) distributed across the frons, labrum, maxillae, galeae, and labium (Fig. 5A). Papilla sensilla occur in all these regions, whereas dome and rod sensilla are restricted to the maxillary and labial palps, making the palps the most morphologically diverse sensory structures examined. Mapping receptor-defined neurons onto this anatomy revealed a striking association between sensillum morphology and chemoreceptor class. *Orco*-positive neurons innervate dome sensilla on the maxillary and labial palps, whereas *Ir25a*-positive neurons innervate papilla sensilla throughout the head. Together, these two populations account for receptor-defined innervation of 34 of the 38 sensilla identified by SEM (Table 1). The four rod sensilla remain unassigned and could potentially receive input from IR-, GR-, mechanosensory, or other sensory neurons. Thus, even at the level of a relatively small peripheral sensory system, sensillum morphology corresponds to distinct molecularly defined neuronal populations.

Comparison among larvae of different species provides a useful framework for distinguishing features of chemosensory organization fundamental to larval life. The degree to which such peripheral organization can be resolved is best illustrated in *Drosophila melanogaster*, which has the best-characterized larval chemosensory system among insects. Early anatomical studies used light and electron microscopy to define the major external and internal sensory organs of the larval head, including the dorsal, terminal, ventral, and pharyngeal organs (32). Over subsequent decades, genetic, molecular, physiological, and neuroanatomical studies progressively linked these structures to defined sensory neurons, chemoreceptors, and central circuits (24, 26, 27, 33, 35, 36, 49, 74–80). More recently, volume electron microscopy and serial-section TEM have provided detailed ultrastructural reconstructions of the external and pharyngeal sensory organs, resolving individual sensilla, cuticular pores and dendritic specializations, the number and arrangement of underlying sensory neurons, and their associated support cells (36, 81). In parallel, molecular studies have mapped chemoreceptor expression onto many of these neuronal populations. Canonical OR-dependent olfaction is mediated by only 21 olfactory receptor neurons in each dorsal organ ganglion, whose dendrites innervate the dorsal-organ multiporous dome and whose axons project to distinct glomeruli in the larval antennal lobe (27, 33–35, 75). Additional neurons associated with the dorsal, terminal, and pharyngeal organs express diverse GRs and IRs and mediate other forms of chemosensation (15, 19, 24, 26, 50, 57, 76, 78–80, 82–86). Beyond chemosensation, IRs and GRs also contribute to sensory modalities including temperature and extraocular light sensing (49, 87). Many GR- and IR-expressing gustatory neurons project to the subesophageal ganglion, producing a central anatomical separation between major olfactory and gustatory pathways (88). The relative simplicity of the *Drosophila* larva has therefore made it possible to follow sensory organization with unusual completeness, from sensillum structure and receptor identity to central circuitry and behavior.

Studies in *Manduca sexta* larvae provide an additional precedent particularly relevant to the central organization we observe in *O. biroi* (70). Morphological analyses have identified multiple classes of cephalic sensilla, including campaniform, styloconic, dome, hair and basiconic sensilla, and characterized structural features such as cuticular pores associated with chemosensory function. Cobalt backfills from individual sensory organs and sensilla further showed that peripheral inputs remain substantially segregated according to their anatomical origin. Antennal afferents project to the larval antennal lobe, labral afferents reach the tritocerebrum and include projections crossing the midline, whereas mouthpart-associated afferents project predominantly to the subesophageal ganglion (70). This general organization is also apparent in *O. biroi*: Orco- and Ir25a-associated projections from the labium and maxilla innervate both the subesophageal ganglion and deutocerebral region (Fig. 5B and C). Labral input enters the tritocerebral region and partially crosses the midline through the tritocerebral commissure, whereas antennal Ir25a-positive projections target the posterior deutocerebral region (Fig. 5C). A broadly similar organization is present in *Drosophila* larvae, where olfactory neurons of the dorsal organ project to the larval antennal lobe, while gustatory and ionotropic sensory neurons from other cephalic sensory organs project predominantly to the subesophageal ganglion. One apparent difference is that olfactory input in *Drosophila* projects directly from the dorsal organ to the larval antennal lobe, whereas in *O. biroi*, Orco-associated mouthpart projections travel together with Ir25a-associated projections to the subesophageal ganglion and then extend toward the deutocerebral region. Together, these similarities suggest that the broad segregation of antennal, labral, and mouthpart-derived sensory information into distinct central territories may be a shared feature of larval insect sensory organization, despite species-specific differences in projection patterns.

The honeybee provides a particularly informative comparison because honeybees are phylogenetically closer to ants and their larvae are similarly dependent on brood care. Honeybee larvae are isolated in individual brood cells and are provisioned by workers (74, 75). Although ant larvae do not forage independently and are also provisioned by workers, they are not confined to brood cells and remain in physical contact with workers, other larvae, and other developmental stages (2, 7). Ant larvae therefore occupy a somewhat intermediate position between honeybee larvae and *Drosophila* larvae, which actively forage within their food substrate (73).

Honeybee larval sensory structures were first described more than a century ago (68), but their ultrastructure, receptor composition, and central projections remain much less completely characterized than those of *Drosophila*. The larval antenna is relatively well described and consists of an antennal mound bearing several papilla-like structures (89–93). These show some similarity in morphology and number to the papilla sensilla of the *O. biroi* larval antenna, although the honeybee structures protrude more prominently from the mound. Maxillary and labial palps have also been described, but not at sufficient ultrastructural resolution to permit detailed comparison with the sensilla identified here. The antenna of the honeybee larva is characterized by *Ir25a* expression and a lack of *Orco* expression (65). Transcriptomic analyses of whole honeybee larvae showed very low GR expression and substantially lower expression of other IR coreceptors relative to *Ir25a* (65). Similar to honeybee larvae, *O. biroi* larvae express *Ir25a*, but not *Orco*, in antennal sensory cells. However, unlike honeybee larvae, they also express substantial levels of multiple GRs and other IR coreceptors in the head, often in the same cells.

Larvae also provide a useful point of comparison with adults, particularly in the context of recent work examining differences in chemosensory organization across developmental stages in honeybees (65). Compared with honeybee adults, which use ORs, IRs, and GRs, honeybee larvae may rely more heavily on Ir25a-mediated chemoreception. These observations led to the hypothesis that development within enclosed brood cells, together with direct provisioning by workers, may have reduced the requirement for larval odor-guided behavior (65). A similar developmental shift may occur in *O. biroi*: IRs appear to dominate chemosensation in larvae, whereas adults heavily rely on a greatly expanded OR system. *O. biroi* larvae nevertheless retain a small population of OR-expressing neurons. In adults, more than 500 tuning ORs, most of which are organized in tandem arrays, are expressed across approximately 10,000 olfactory sensory neurons per antenna (39). Receptor choice is governed by a complex mechanism that remains incompletely understood but involves bidirectional transcription, transcriptional readthrough, and post-transcriptional regulation through differential nuclear export (37, 38). Interestingly, among the ORs examined, those detected exclusively in Orco-positive sensory neurons were singleton genes located outside the major OR tandem arrays. Although only a small subset of ORs was examined, this pattern raises the possibility that tuning OR expression may differ between larval and adult olfactory systems.

Together, our results establish an anatomical and molecular framework for the *O. biroi* larval chemosensory system, extending from external sensilla to receptor-defined sensory neurons and their central projections (Fig. 5A–C). Comparisons with *Drosophila*, *Manduca*, and honeybee larvae point to several shared features of larval chemosensory organization, including a prominent role for IRs in the two hymenopteran systems and broad similarities in the central segregation of sensory inputs. At the same time, they reveal species-specific differences in receptor expression and projection patterns. These differences may in part reflect variation in larval ecology and behavior. Larval insect chemosensory systems therefore offer not only simplified versions of adult systems, but comparative models for understanding how sensory circuits are adapted to the chemical, ecological, and social environments experienced during development.

## Materials and Methods

### Ant husbandry and maintenance

*O. biroi* larvae were obtained from large stock colonies maintained at 25 °C in 32 × 14 cm plastic containers with a plaster of Paris floor. During the foraging phase, colonies were fed frozen fire ant (*Solenopsis invicta*) brood three times per week and cleaned and watered weekly as needed.

For scanning electron microscopy, larvae were aged and assigned to instars based on Dyar’s law, grouping larvae according to the increase in head capsule width at each molt (94). In this staging scheme, first and second instars corresponded to the first three days post hatching, third instars to four to six days post hatching, and fourth instars to six to fourteen days post hatching.

For all other experiments, larvae were collected 2–5 days after the transition from the third to the fourth instar based on visual inspection. All animals were derived from clonal line B.

### Scanning electron microscopy

Larvae were fixed in 2% glutaraldehyde and 4% paraformaldehyde in 0.1 M sodium cacodylate buffer, pH 7.2, for 3 h at room temperature, and overnight at 4 °C. After rinsing three times with cacodylate buffer, samples were dehydrated in a graded ethanol series (30%, 50%, 70%, 90%) for 10 min each, then three times in 100% ethanol with molecular sieves for 15 min each, and finally critical point dried in a Tousimis Autosamdri 931. Samples were imaged on a JEOL JSM-IT500HR scanning electron microscope at 5.0 kV. For Cryo-SEM, larvae were frozen in liquid nitrogen (LN2) and mounted on a cryo-stub (JEOL, Cat. JU2010535) precooled to LN2 temperature. Samples were imaged within 30 min on a JEOL JSM-IT500HR scanning electron microscope under low-vacuum mode (50 Pa) at 20 kV.

### Immunohistochemistry

Antibody staining was performed on larval heads dissected from fourth-instar larvae. Heads were separated from the body at the head–thorax junction, anterior to the point where the subesophageal ganglion transitions into the ventral nerve cord. Dissections were carried out in cold phosphate-buffered saline containing Tween 20 (PBST). Samples were fixed in 4% paraformaldehyde (PFA) for one hour at room temperature.

Following fixation, tissues were blocked for one hour in freshly prepared blocking buffer containing 0.5% Triton X-100 and 5% normal donkey serum. Samples were then incubated with primary antibodies diluted in fresh blocking buffer and placed on an orbital shaker at room temperature.

After primary incubation, tissues were washed in PBST three times for five minutes each and incubated with fluorophore-conjugated secondary antibodies diluted in fresh blocking buffer. Following secondary antibody incubation, samples were washed three times in PBST for 5 min each and prepared for mounting.

Primary antibodies were chicken polyclonal anti-GFP (Abcam, Cat. no. ab13970, RRID: AB_300798; 1:1,000) and mouse monoclonal anti-Orco (clone 20F7; gift from Vanessa Ruta; 1:200). Secondary antibodies were goat anti-chicken IgY conjugated to Alexa Fluor 488 (Invitrogen, Cat. no. A-11039, RRID: AB_2534096), Alexa Fluor 594 (Invitrogen, Cat. no. A-11042, RRID: AB_2534099), or Alexa Fluor 647 (Invitrogen, Cat. no. A-21449, RRID: AB_2535866), each used at 1:100, and donkey anti-mouse IgG Alexa Fluor Plus 647 (Invitrogen, Cat. no. A32787, RRID: AB_2762830; 1:200). For some experiments, DAPI was included during secondary antibody incubation. Stained tissues were mounted in SlowFade Glass mounting medium on frosted microscope slides.

### RNA-FISH staining

Sample dissection for RNA-FISH was performed similarly to immunohistochemistry. Larval heads were separated and fixed in 4% PFA for one hour at room temperature, then washed three times for five minutes each in PBST.

Samples were dehydrated through a graded methanol series (25%, 50%, 75% methanol in PBST), followed by two ten-minute washes in 100% methanol. Samples were stored in 100% methanol at −20 °C for at least 24 hours and up to six months.

RNA-FISH probes were designed as described previously (33), ordered from Integrated DNA Technologies (IDT) or Molecular Instruments (MI), and diluted to 1 µM stocks.

Prior to hybridization, samples were rehydrated on ice through a reverse methanol series (75%, 50%, 25% in PBST), followed by two ten-minute washes in PBST. Hybridization buffer was warmed to 37 °C. Samples were incubated in 500 µL hybridization buffer for five minutes, then transferred to probe-containing buffer (3 µL of 1 µM probe in 250 µL buffer) and incubated at 37 °C for 16 to 48 hours. After hybridization, samples were washed four times for 15 minutes each in prewarmed probe wash buffer at 37 °C, then two times for five minutes each in 5× SSCT at room temperature. Samples were stored in SSCT for up to 16 hours or processed immediately.

For amplification, 6 µL of each hairpin was heated at 95 °C for ninety seconds and cooled for thirty minutes in the dark. Samples were incubated in amplification buffer for five minutes, then in amplification mix (100 µL amplification buffer with 0.3 µL DAPI and combined hairpins) for 16 hours at room temperature with rotation at 300 rpm in the dark.

Following amplification, samples were washed two times for five minutes each in SSCT, then two times for thirty minutes each in the dark and mounted in SlowFade Glass mounting medium.

### Confocal microscopy

Larval heads stained by IHC or RNA-FISH were imaged on a Zeiss LSM 900 laser-scanning confocal microscope using 405-, 488-, 561-, and 640-nm excitation. Z-stacks were acquired using a Zeiss LD LCI Plan-Apochromat 40×/1.2 NA multi-immersion objective with glycerol immersion and ZEN Blue 3.5 software (Zeiss). Channels were acquired sequentially, frame by frame, with the confocal pinhole set to 1 Airy unit. Images were typically acquired at 2,048 × 2,048 pixels with a 0.5-µm z-step; acquisition parameters were adjusted when necessary, according to the field of view and imaging scale. Laser power and detector settings were adjusted for each staining experiment to obtain adequate signal while avoiding pixel saturation.

### Cell Counts

Custom code was used to assist with cell counting in FISH and IHC images, although equivalent counts could also be obtained using FIJI. Cells were considered positive when a minimum of three puncta were present within or immediately surrounding the cell. Some discretion was applied in cases where nearby cuticular structures exhibited autofluorescence or where signal from an intensely positive neighboring cell interfered with interpretation.

### Two-photon microscopy

Two-photon imaging was performed on a Bruker Investigator two-photon laser-scanning microscope (Bruker) equipped with galvanometer and resonant scanning pathways and a Coherent Axon laser tuned to 920 nm. Images were acquired using Prairie View software (Bruker) with an Olympus 40×/0.8 NA objective. Images were acquired at either 1,024 × 1,024 or 2,048 × 2,048 pixels. Laser power and detector gain were adjusted to obtain adequate fluorescence signal without saturation. Two-photon microscopy was used to visualize sensillar innervation by *Ir25a*-GCaMP–expressing cells (Fig. 3B–H).

### Transgenic line generation

The transgenic *O. biroi* line expressing GCaMP7s under an *Ir25a* promoter fragment was generated by piggyBac transposition as described previously (71) using the hyperactive piggyBac variant hyPBase^apis^. The piggyBac donor vector, pBAC-ie1-DsRed-ObirIr25a.2prom1-QF2–5xQUAS-GCaMP7s-T2A-tdTomato, was designed by the authors and then synthesized by VectorBuilder (Vector ID: VB240410–2037jjg; VectorBuilder Inc., Chicago, IL, USA). ObirIr25a.2prom1 is a 2-kb promoter/enhancer fragment, including intergenic sequence and the entire *Ir25a* 5’ UTR based on the clonal raider ant genome (NCBI LOC105280757; chromosome 8: 11792544–11794543) (Fig. S6A).

Microinjections were performed as described previously (95), with the following modifications: Eggs from *O. biroi* clonal line B were injected into the posterior end and not taped. After injection, they were reared in small colonies by adult *O. biroi* workers from clonal line A. G1 individuals were reared as described previously (71) to ensure the generation of an isogenic population in which all ants are derived from a single founder individual and share the same transgene insertion.

Genomic DNA from a single transgenic individual was extracted using the Qiagen Low Input Kit and subjected to whole-genome resequencing. Libraries were prepared using the KAPA HyperPrep method and sequenced on an Element AVITI using a 500M flow cell with 150-bp paired-end reads.

Reads were aligned to the *O. biroi* reference genome (Obir_v5.4; GenBank assembly GCA_003672135.1) and the transgene sequence, and transgene copy number and insertion sites were determined using previously described read-depth and transgene–genome junction-read analyses (71, 96). Copy number was estimated by comparing sequencing depth at heterozygous genomic SNPs with that across the *Ir25a.2* promoter and other regions of the transgene. Two transgene insertions were identified, one on chromosome 1 at position 23,273,490 within an intron of *LOC105283447*, which encodes TAK1-associated binding protein 2 (Tab2), and one on chromosome 11 at position 8,873,437 in an intergenic region (Fig. S6B–E).

### RNA sequencing

#### Sample preparation

Four independent genotype B colonies were established, each containing 25 one-month-old caregiver workers and 25 first-instar larvae housed in 50 mm Petri dishes. Colonies were maintained at 27 ± 1 °C and fed every 24 h.

Larvae were collected 12–24 h after at least 50% of individuals in a colony had reached the final larval instar and immediately before the daily feeding. Heads were removed at the posterior border of the prothorax using microdissection scissors. Each biological replicate consisted of ten pooled larval heads from a single colony.

#### RNA extraction

Larval heads were dissected directly into TRIzol Reagent (Thermo Fisher Scientific) on ice, flash-frozen on dry ice, and stored at −80 °C. Samples were thawed on ice and homogenized in TRIzol using a TissueLyser II (QIAGEN) with stainless steel beads for 2 min at 30 Hz. Samples underwent one freeze–thaw cycle on dry ice followed by a second 2-min homogenization at 30 Hz. Homogenates were centrifuged at 21,300 × *g* for 1 min, and lysates were transferred to Heavy Phase Lock Gel tubes (Quantabio). Chloroform (Sigma-Aldrich, C2432) was added at 0.2 mL per 1 mL TRIzol. Samples were shaken vigorously for 15 s, incubated for 2 min at room temperature, and centrifuged at 14,000 × *g* for 10 min at 4 °C. The aqueous phase was mixed with an equal volume of 70% ethanol and applied to an RNeasy Mini spin column (QIAGEN). RNA was purified according to the manufacturer's instructions, including on-column DNase digestion with RNase-Free DNase (QIAGEN). RNA concentration and integrity were assessed using an Agilent 2100 Bioanalyzer.

#### RNA sequencing and read quantification

Library preparation and sequencing were performed by the Genomics Resource Center at The Rockefeller University (RRID:SCR_020986). cDNA was generated from 1 ng total RNA using the SMARTer Stranded Total RNA-Seq Kit (Takara Bio) with rRNA depletion, and sequencing libraries were prepared using the Nextera XT DNA Library Preparation Kit (Illumina). Fragment size distributions were assessed using an Agilent High Sensitivity D1000 ScreenTape Assay, with an average insert size of ~300 bp excluding ~120 bp adaptor sequence. Libraries were multiplexed and sequenced in a single lane on an Illumina NovaSeq 6000 using an S2 flow cell to generate 2 × 100-bp paired-end reads.

Reads were quality filtered and adapter trimmed using fastp through the Rfastp wrapper (97) and aligned to the *O. biroi* reference genome (v5.4; GenBank GCA_003672135.1) using subjunc in Rsubread (98). Gene-level counts were generated from BAM files using featureCounts in Rsubread (98), with exon features defined by the _O. biroi_ OGS5.2 GTF annotation deposited on Zenodo. Counts were converted to transcripts per million (TPM) and used to rank odorant receptors, ionotropic receptors, and gustatory receptors by expression level.

#### Use of generative AI

ChatGPT (OpenAI) was used to assist with grammar, syntax, wording, and formatting during manuscript preparation. OpenAI Codex was also used to assist with generation and refinement of custom code used for cell counting and figure assembly. Generative AI was not used to generate scientific hypotheses, interpret experimental results, or determine the conclusions of the study. All AI-assisted text and code was reviewed and verified by the authors, who take full responsibility for the content of the manuscript and analyses.

## Supplementary Material

Supplement 1

## Figures and Tables

**Fig. 1.**
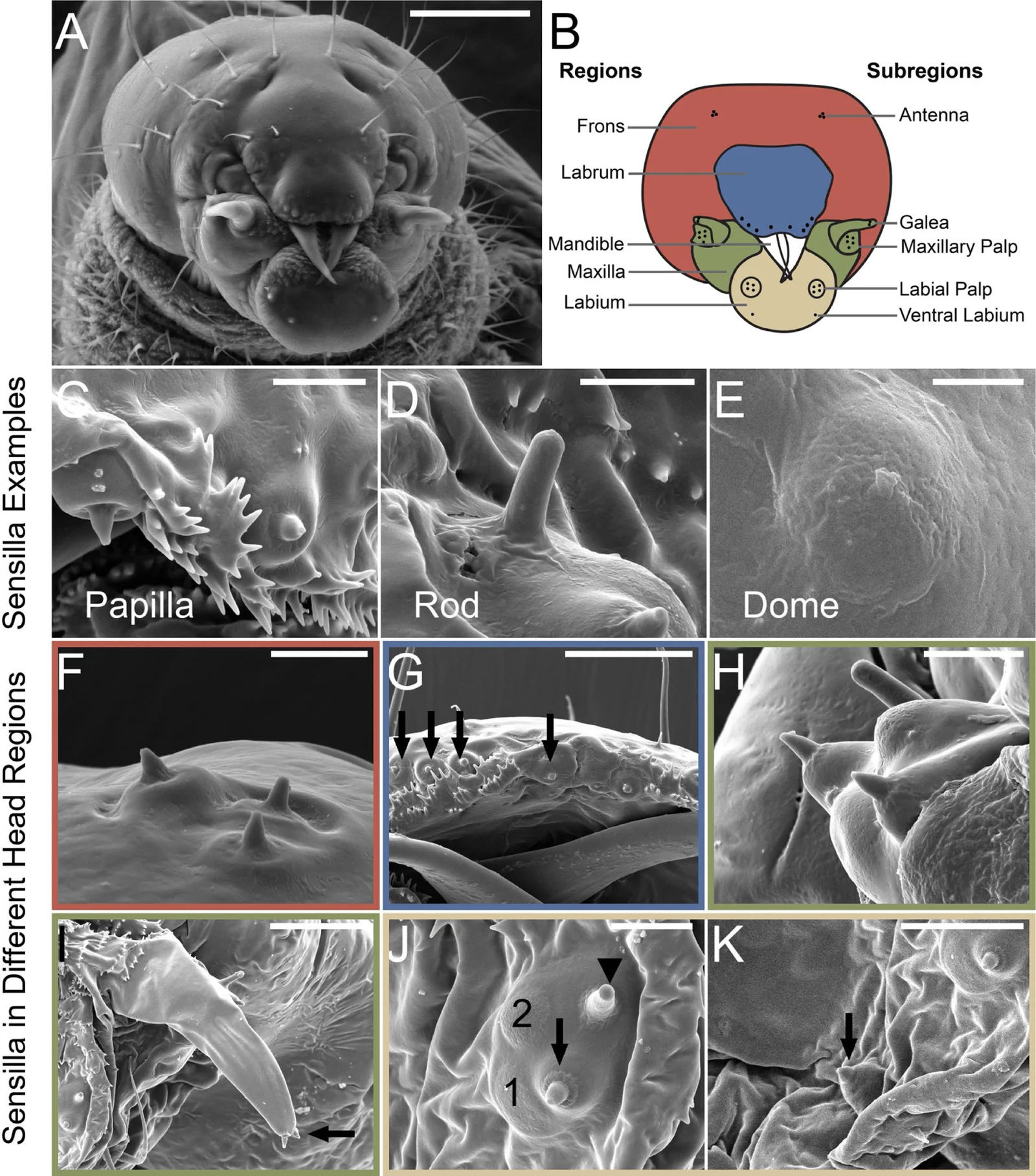
Sensory anatomy of the *Ooceraea biroi* larval head. (A) Cryo-scanning electron micrograph of the head of a fourth-instar larva showing the major sensory organs analyzed in this study. (B) Schematic of the image in (A) showing the head regions and subregions. Black dots denote individual sensilla; colors correspond to the panel borders in F–K and are used consistently throughout the paper. (C–E) Representative examples of the three sensillum types identified on the larval head: papilla (C), rod sensillum (D), and dome sensillum (E). (F–K) High-magnification views of sensory organs arranged from dorsal to ventral: antennal region, with three papillae per side (F); labrum, with four papillae per side (G); maxillary palp, with two papillae, two dome sensilla, and one rod sensillum (H); galea, with two distal papillae (I); labial palp, with one papilla, one rod sensillum, and two dome sensilla (J); and ventral labial region, with a single papilla (K). Black arrows mark partially obscured papillae. In J, the arrowhead marks a rod sensillum viewed from above, and numerals 1 and 2 identify dome sensilla viewed from above. Images in C–K were obtained by conventional SEM and are representative of two fourth-instar larvae. Scale bars: 80 μm (A); 5 μm (C, F, H, and J); 4 μm (D); 2 μm (E); 20 μm (G and I); and 10 μm (K).

**Fig. 2.**
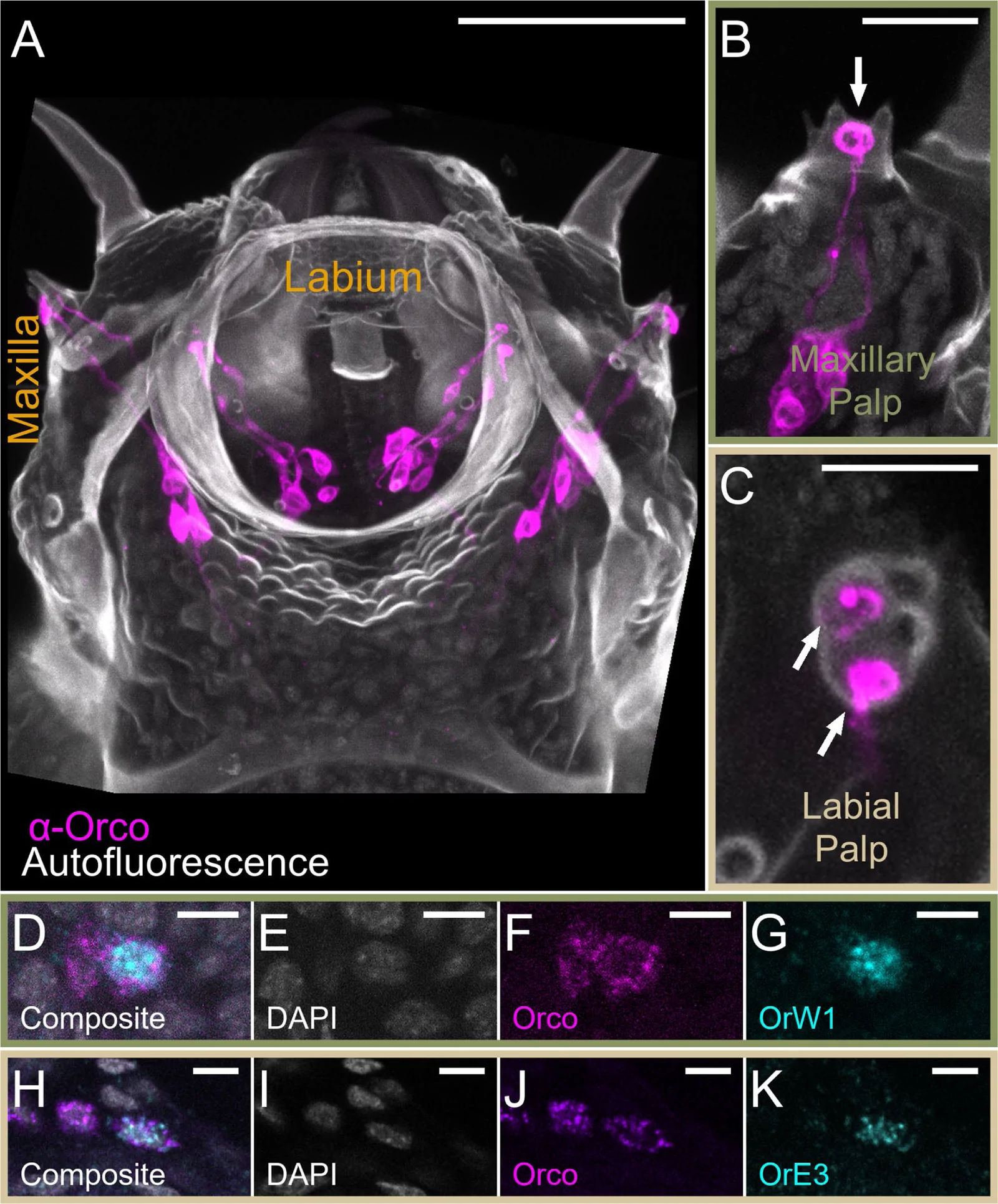
Odorant receptor expression in the mouthparts of fourth-instar *Ooceraea biroi* larvae. (A) Overview of Orco-expressing sensory neurons visualized by anti-Orco immunostaining (magenta); cuticular autofluorescence in the DAPI channel is shown in white. (B) Magnified view of the maxillary palp showing neurons innervating the dome sensilla. (C) Magnified view of the labial palp showing neurons innervating the dome sensilla. (D–G) *OrW1* expression in the maxilla of a wild-type fourth-instar larva, shown as a composite image and as the individual DAPI, Orco, and *OrW1* channels. (H–K) *OrE3* expression in the labium of a wild-type fourth-instar larva, shown as a composite image and as the individual DAPI, Orco, and *OrE3* channels. Arrows in B and C indicate the dome sensilla. In B, the two dome sensilla are located between the papillae; one is visible in the focal plane, whereas the second lies behind it. Scale bars: 40 µm (A); 20 µm (B); 10 µm (C); 5 µm (D–K).

**Fig. 3.**
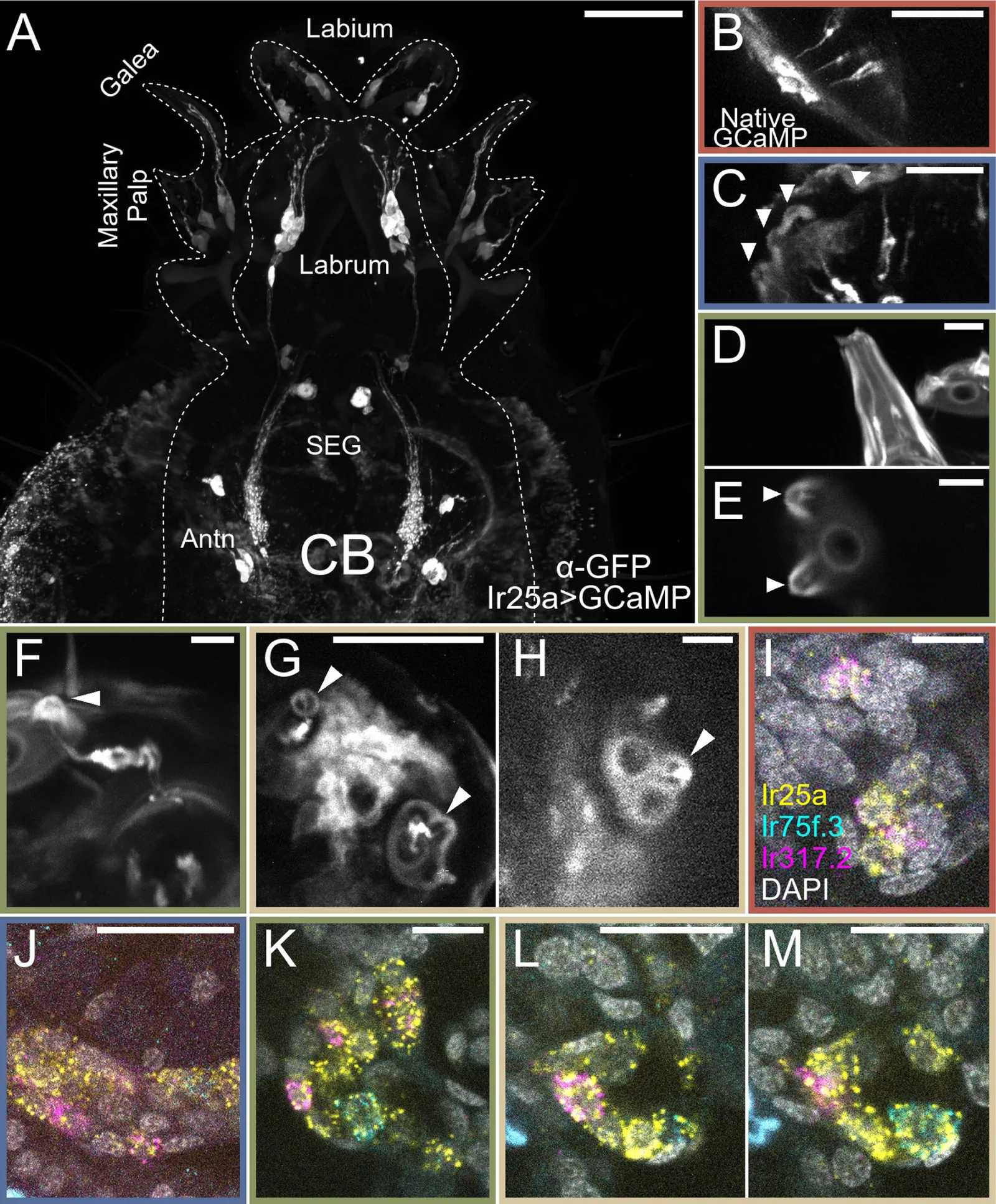
Organization of *Ir25a*-positive sensory neurons and ionotropic receptor expression in the fourth-instar *Ooceraea biroi* larval head. (A) Maximum-intensity z-projection of the larval head showing GCaMP expression driven by an *Ir25a* promoter fragment. (B–H) GCaMP-positive neurons innervating papillae in the antennal region (B), labrum (C), galea (D), maxillary palp (E and F), labial palp (G), and ventral labial region (H). Three papillae are innervated in the antennal region (B), four on one side of the labrum (C), two in the galea (D), and two in the maxillary palp (E and F). (F) shows a different view of one papilla. Arrowheads indicate papillae in (C) and (E-H). (I–M) RNA-FISH detection of *Ir317.2* and *Ir75f.3* in *Ir25a*-positive cells. *Ir317.2*, but not *Ir75f.3*, was detected in the antennal region (I), whereas both receptors were detected in the labral (J), maxillary (K), and labial regions (L and M). DAPI counterstain is shown in white. Scale bars: 100 µm (A); 25 µm (B); 10 µm (C, E, F, and K); 15 µm (D, J, L, and M); 20 µm (G); and 5 µm (H and I).

**Fig. 4.**
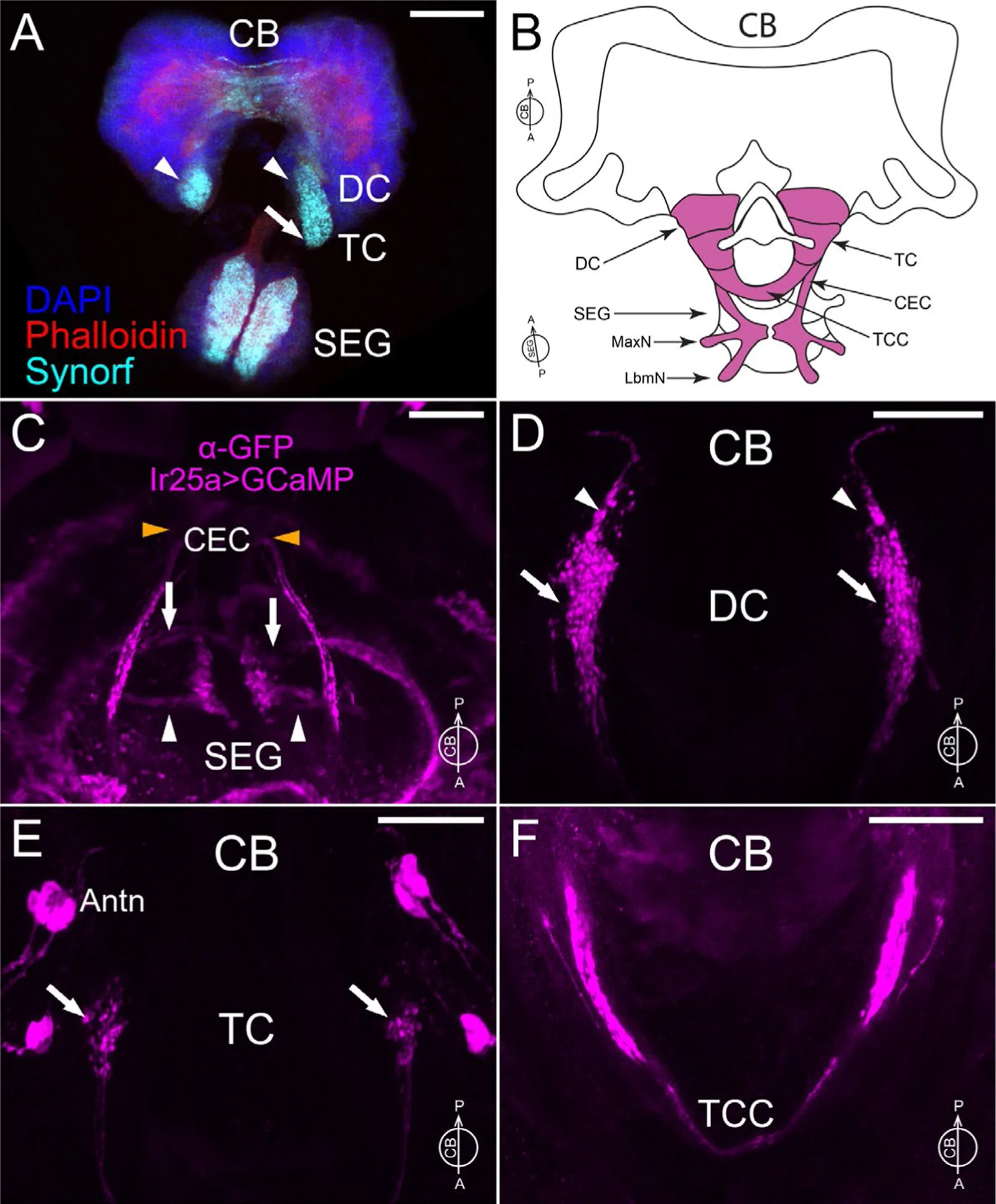
Central projections of *Ir25a*-positive sensory neurons in the *Ooceraea biroi* larva. (A) Overview of the central brain (CB) and subesophageal ganglion (SEG) from a third-instar larva. The arrowheads and arrow indicate the approximate locations of the deutocerebrum (DC) and tritocerebrum (TC), respectively. DAPI labels nuclei, phalloidin labels F-actin-rich structures, and anti-Synorf1 labels synapse-rich neuropil. (B) Schematic of the brain regions containing GCaMP signal from *Ir25a*-positive sensory neurons, corresponding to C–F. For clarity, the central brain and SEG are shown perpendicular to one another, although they are positioned approximately parallel to each other within the head. Compass symbols indicate their anatomical orientation in situ. CEC, circumesophageal connective; MaxN, maxillary nerve; LbmN, labial nerve; TCC, tritocerebral commissure. (C) GCaMP signal in the maxillary and labial nerves (white arrows and arrowheads, respectively), which carry *Ir25a*- and *Orco*-associated sensory input from the mouthparts through the SEG and into the central brain via the CECs (orange arrowheads). (D) GCaMP-positive projections from *Ir25a* neurons in the maxillary and labial nerves enter the DC via the SEG. These projections are shown here in the deutocerebral region (arrows). GCaMP-positive *Ir25a* neurons in the antenna also project to the DC, but to a more posterior region (arrowheads). (E) GCaMP-positive projections in the tritocerebral region associated with labral sensory neurons expressing *Ir25a* but not *Orco* (arrows). Antennal neurons are located nearby but do not innervate the TC. Their projections are not shown here. One antennal neuron is labelled Antn as an example. (F) GCaMP-positive fibers connecting the left and right tritocerebral regions through the tritocerebral commissure. Scale bars: 50 µm (A) and 25 µm (C–F). Panels (C-F) show fourth-instar larvae.

**Fig. 5.**
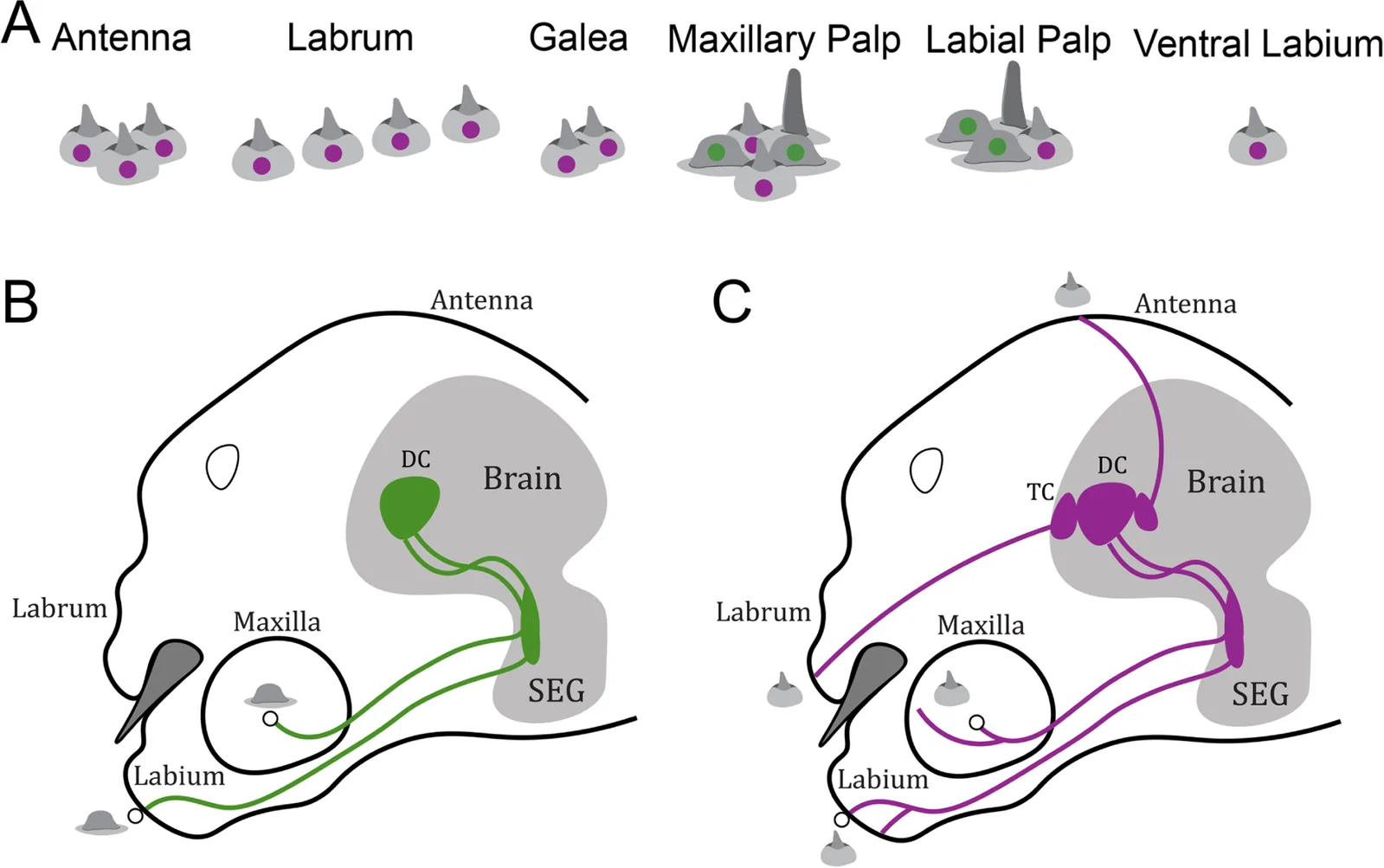
Peripheral organization and central projections of larval chemosensory pathways. (A) Summary of the spatial organization of the three sensillum types (papilla, rod, and dome) within different regions of the larval head, as well as their distribution across different regions (structures are paired across the two sides of the head and are shown here for one side). Green dots indicate innervation by odorant receptor neurons (ORNs; Orco-positive) and purple dots indicate innervation by ionotropic receptor neurons (IRNs; IR25a-positive). (B) Schematic of ORN projections from dome sensilla in the maxilla and labium. These projections travel through the subesophageal ganglion (SEG), where they synapse, and continue to the deutocerebrum (DC) in the central brain. (C) Schematic of IRN projections from papilla sensilla. Antennal projections terminate in the posterior DC, labral projections terminate in the tritocerebrum (TC), and maxillary and labial projections travel through the SEG, where they also synapse, and continue to the DC. Open circles in (B) and (C) indicate the maxillary and labial palps.

**Table 1.** Distribution of chemosensory sensilla across the clonal raider ant larval head.

Region	Subregion	Sensilla

Frons	Antennae	Papilla (6)
Labrum		Papilla (8)
Maxilla	Maxillary palps	Dome (4)
		Papilla (4)
		Rod (2)
	Galeae	Papilla (4)
Labium	Labial palps	Dome (4)
		Papilla (2)
		Rod (2)
	Ventral labium	Papilla (2)

Numbers in parentheses indicate the total number of each sensillum type observed across the entire head (left and right sides) of fourth-instar larvae using scanning electron microscopy (n = 2 biological replicates).

## Data Availability

Custom code used for cell counting and figure assembly is available at the project GitHub repository: https://github.com/Social-Evolution-and-Behavior/Larval-Chemosensory-Paper-2026. Raw paired-end RNA-sequencing reads generated in this study are available on Zenodo at doi: 10.5281/zenodo.21922646.
