# Supplementary material for: The chemosensory system of the clonal raider ant larva
Source: bioRxiv. 2026 Oct 9:2026.10.06.757048. Preprint. [Version 1] doi: 10.64898/2026.10.06.757048 (PMC13692247; doi:10.64898/2026.10.06.757048)
Supplement: Supplementary file 1 [file media-1.pdf]

Supporting Information for:

The chemosensory system of the clonal raider ant larva

P. Daniel H. Pastor[1,2,*], Leonora Olivos-Cisneros[2], Alejandra Hurtado-Giraldo[2], Anurag Sharma[3], Patrick K. Piekarski[2], Giacomo L. Glotzer[2], Daniel J. C. Kronauer[2,4,*]

1 Tri-Institutional MD–PhD Program, Weill Cornell/Rockefeller/Sloan Kettering, New York, NY 10065, USA

2 Laboratory of Social Evolution and Behavior, The Rockefeller University, New York, NY 10065, USA

3 Electron Microscopy Resource Center, The Rockefeller University, New York, NY 10065, USA

4 Howard Hughes Medical Institute, New York, NY 10065, USA

*Corresponding authors: P. Daniel H. Pastor and Daniel J.C. Kronauer

Email: phejazi@rockefeller.edu and dkronauer@rockefeller.edu

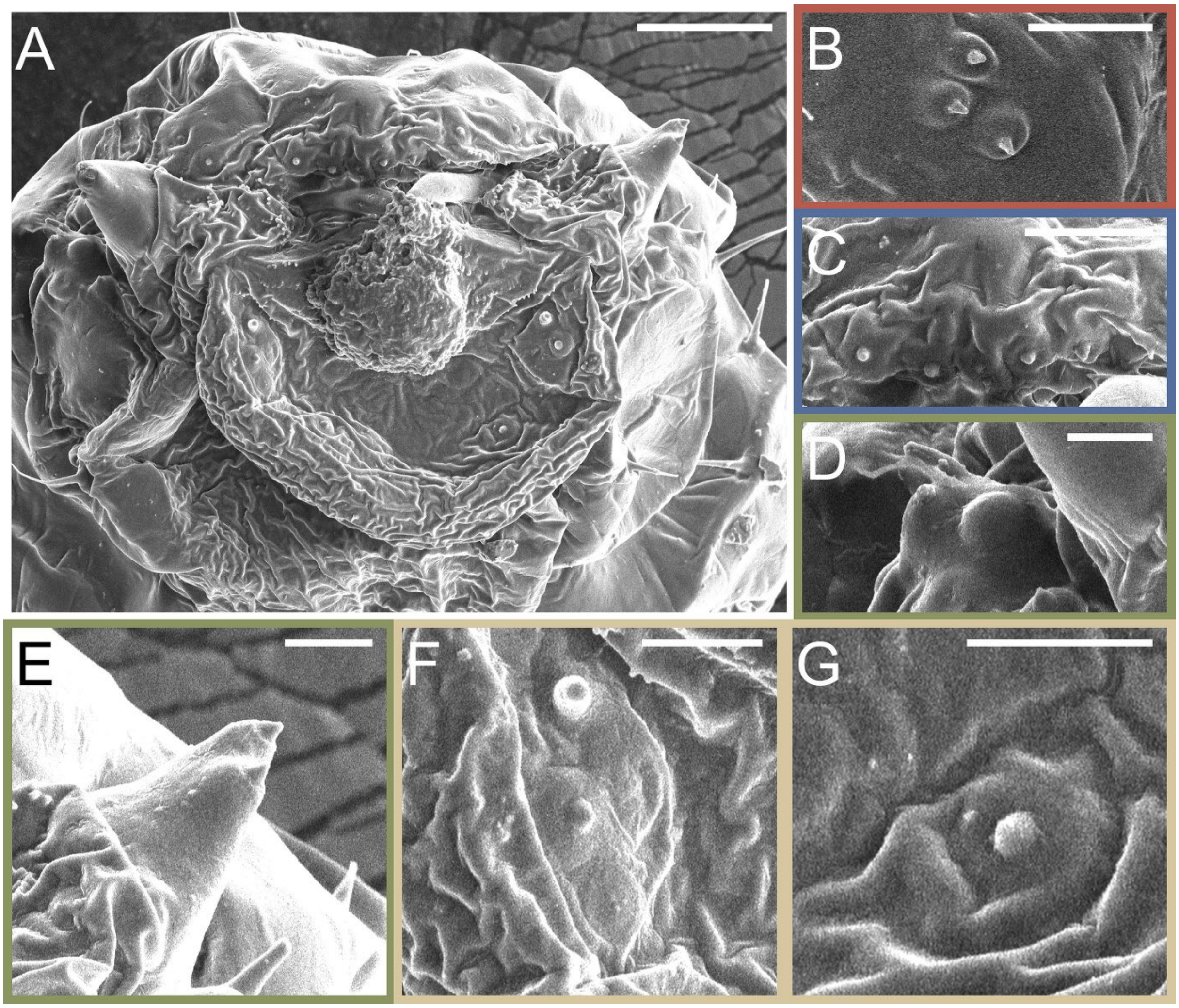

Fig. S1. Sensory anatomy of early-instar *Ooceraea biroi* larvae. (A) Scanning electron micrograph of the head of a first- or second-instar larva showing the major sensory organs. (B–G) Higher-magnification views of the antenna (B), labrum (C), maxillary palp (D), galea (E), labial palp (F), and ventral labium (G). Scale bars: 20 µm (A); 10 µm (B and C); and 5 µm (D–G). Images were obtained from first- and second-instar larvae. Border colors correspond to the anatomical regions defined in Fig. 1B.

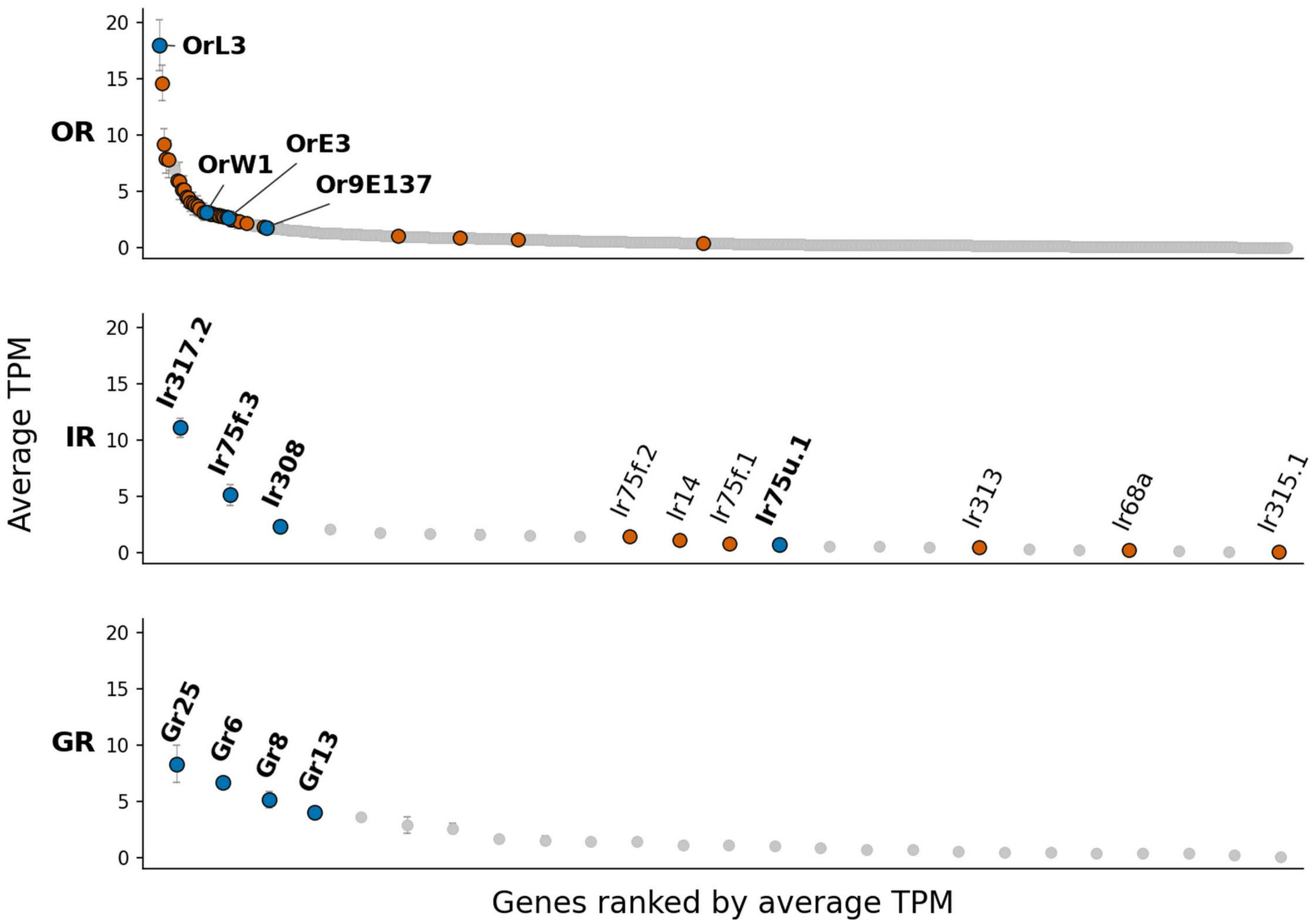

Fig. S2. Bulk RNA-seq expression of chemosensory receptor genes in clonal line B fourth-instar *Ooceraea biroi* larval heads. Average transcript abundance of odorant receptor (OR), ionotropic receptor (IR), and gustatory receptor (GR) genes. Within each receptor family, genes are ranked from highest to lowest average transcripts per million (TPM). Blue circles indicate genes detected by RNA-FISH, orange circles indicate genes tested but not detected, and gray circles indicate genes not tested. The two tuning ORs detected by RNA-FISH, *E3* and *W1*, were not among the most highly expressed OR genes in the bulk RNA-seq dataset. Labels identify genes tested by RNA-FISH. Error bars indicate SEM.

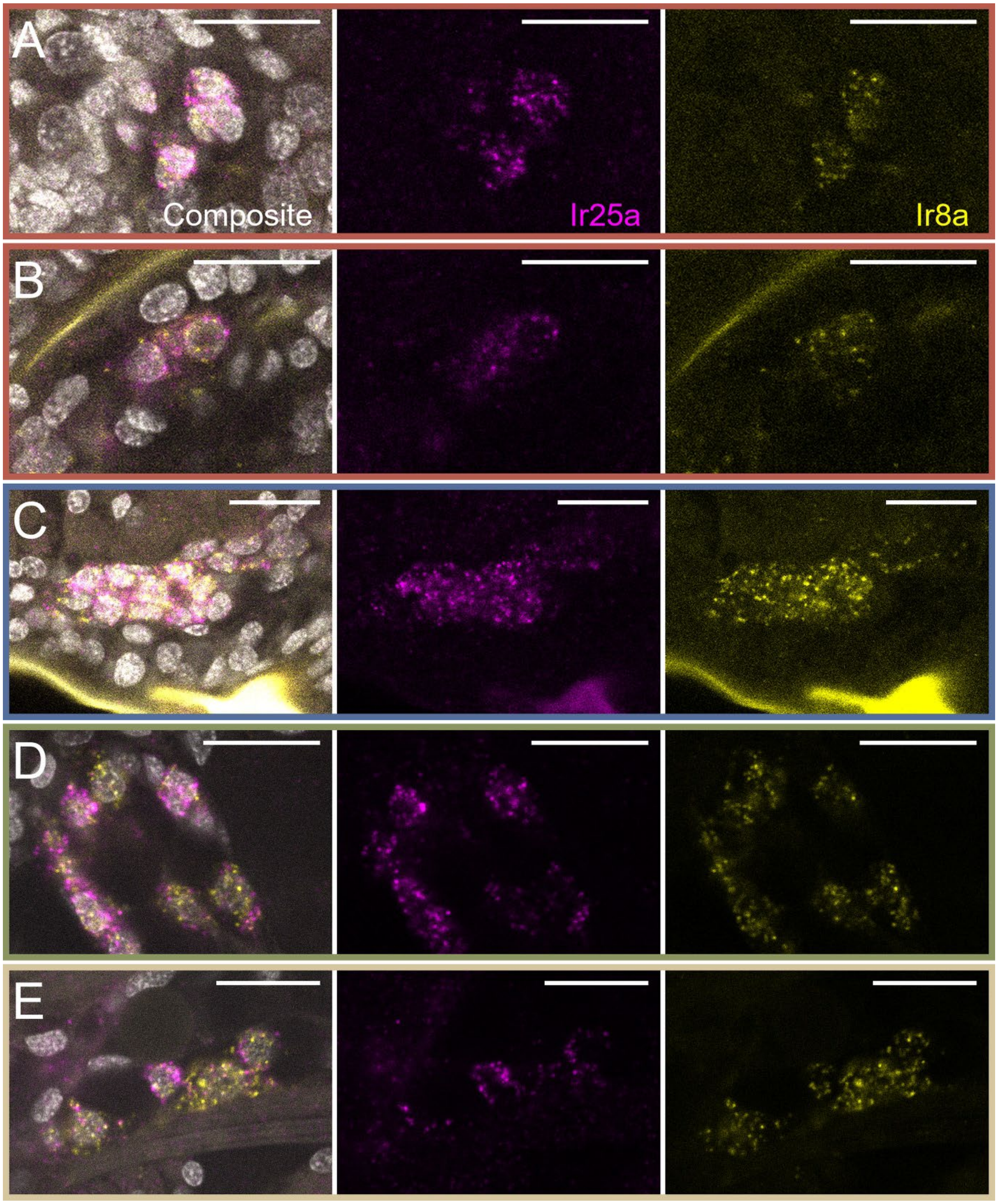

Fig. S3. Widespread coexpression of *Ir25a* and *Ir8a* in larval head sensory organs. (A–E) RNA-FISH detection of *Ir25a* (magenta) and *Ir8a* (yellow) in sensory neurons of the antennal (A and B), labral (C), maxillary (D), and labial (E) regions. The two coreceptors are coexpressed in many cells, although their relative signal intensities vary among regions. DAPI is shown in white in the composite images. Border colors correspond to the anatomical regions defined in Fig. 1B. Scale bars: 15 μm.

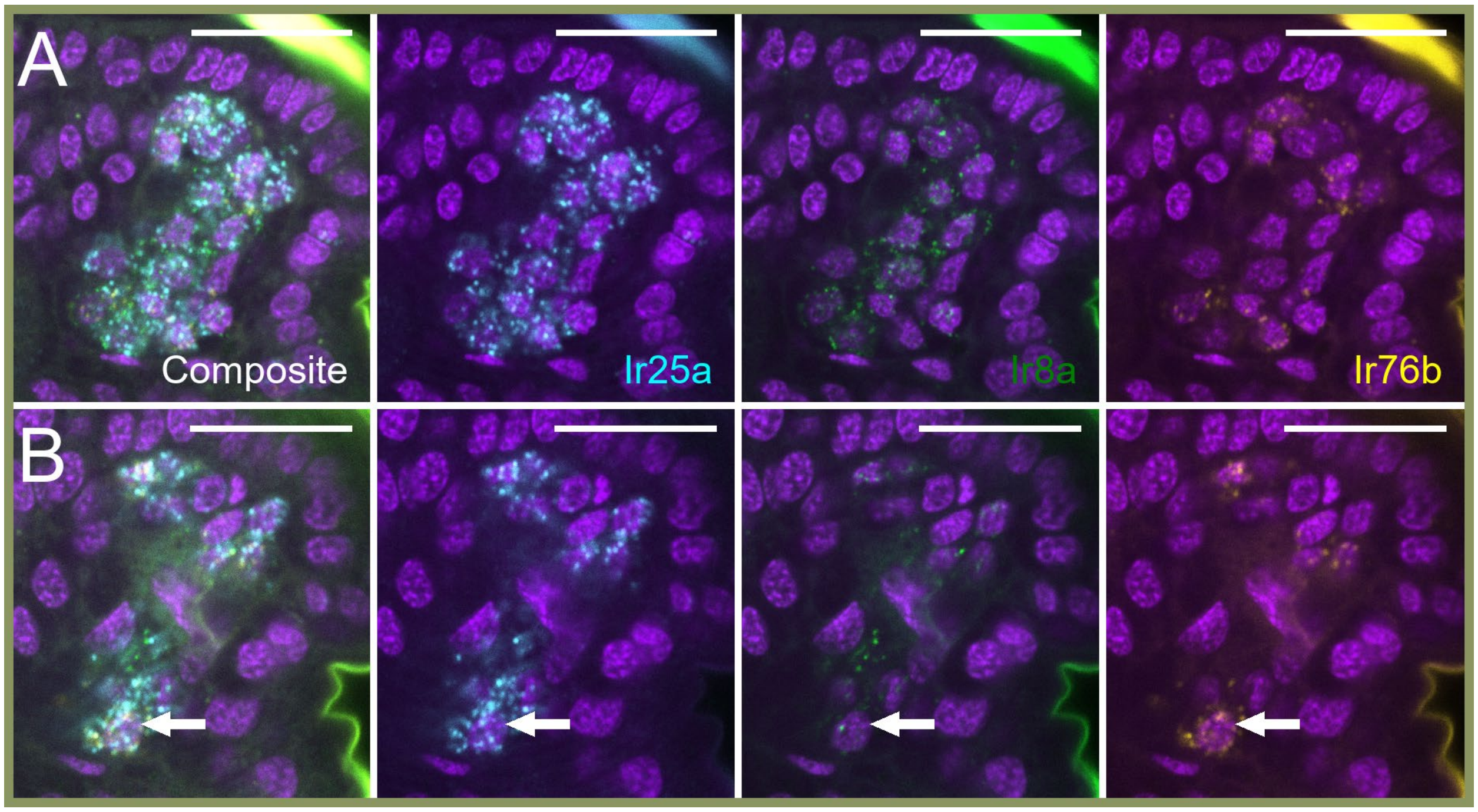

Fig. S4. Combinatorial coexpression of *Ir25a*, *Ir8a*, and *Ir76b* in the larval maxilla. Representative RNA-FISH images showing expression of *Ir25a* (cyan), *Ir8a* (green), and *Ir76b* (yellow) in maxillary sensory cells. *Ir25a* was detected in the broadest cell population, followed by *Ir8a*, whereas *Ir76b* was restricted to a smaller subset. Some cells coexpressed all three coreceptors, whereas others coexpressed *Ir25a* with either *Ir8a* or *Ir76b*. Arrows in (B) mark an example of *Ir25a* and *Ir76b* coexpression with little detectable *Ir8a*. DAPI is shown in magenta. Scale bars: 15 μm.

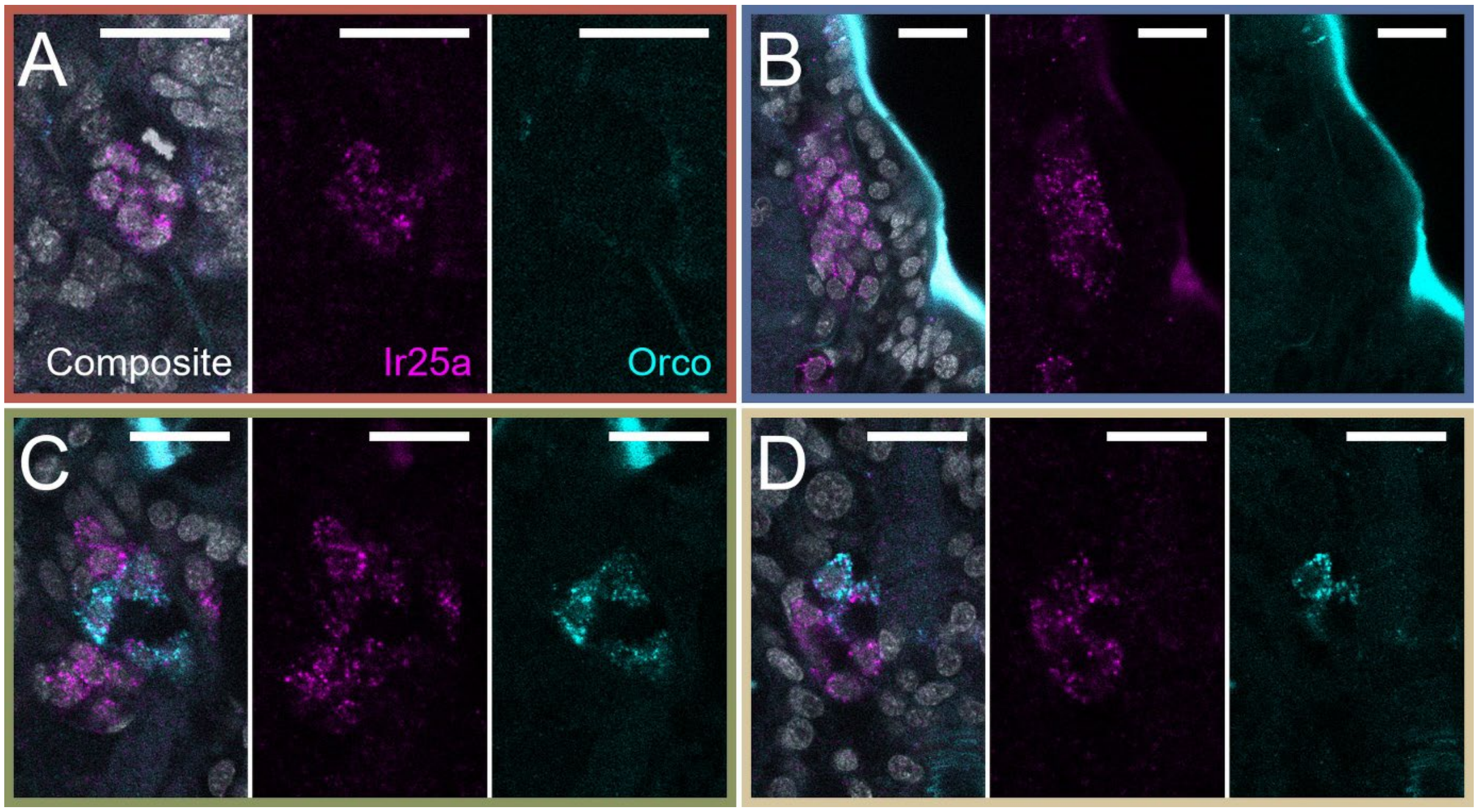

Fig. S5. Largely nonoverlapping expression of *Ir25a* and *Orco* in larval head sensory organs. (A–D) RNA-FISH detection of *Ir25a* (magenta) and *Orco* (cyan) in the antennal (A), labral (B), maxillary (C), and labial (D) regions. The two genes are expressed in largely nonoverlapping cell populations, although faint *Ir25a* signal is detectable in some *Orco*-positive cells. DAPI is shown in white in the composite images. Border colors correspond to the anatomical regions defined in Fig. 1B. Scale bars: 15 μm.

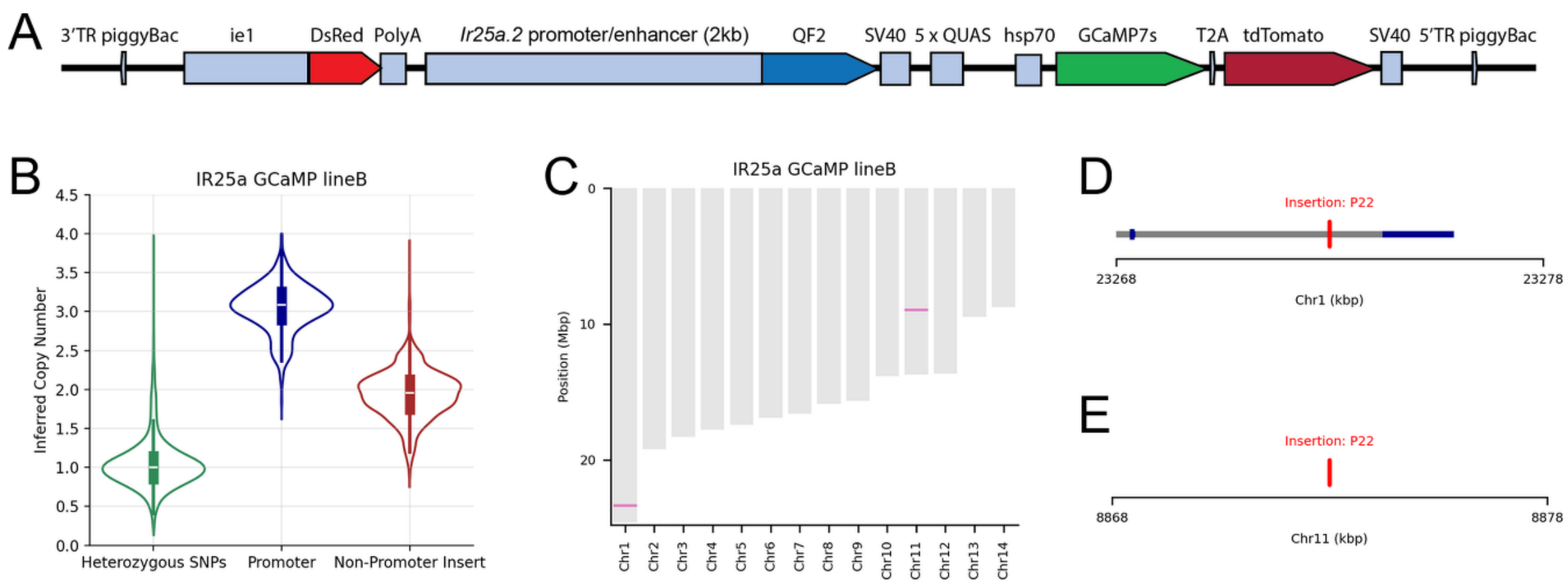

Fig. S6. Generation and genomic characterization of the *Ir25a*-GCaMP transgenic line. (A) Schematic of the piggyBac donor construct used to generate the *Ir25a*-GCaMP transgenic line, showing the major regulatory and coding elements. (B) Inferred transgene copy number based on whole-genome sequencing coverage of heterozygous SNPs, the *Ir25a.2* promoter fragment, and non-promoter regions of the transgene. (C–D) Genomic mapping of transgene insertion sites by whole-genome sequencing. (C) Chromosomal positions of the two identified piggyBac insertions (magenta), located on chromosomes 1 and 11. (D and E) Genomic context of the chromosome 1 and chromosome 11 insertion sites, respectively. Insertion on chromosome 1 is within an intron of *LOC105283447*, which encodes TAK1-associated binding protein 2 (Tab2). The red marker indicates the mapped insertion position.

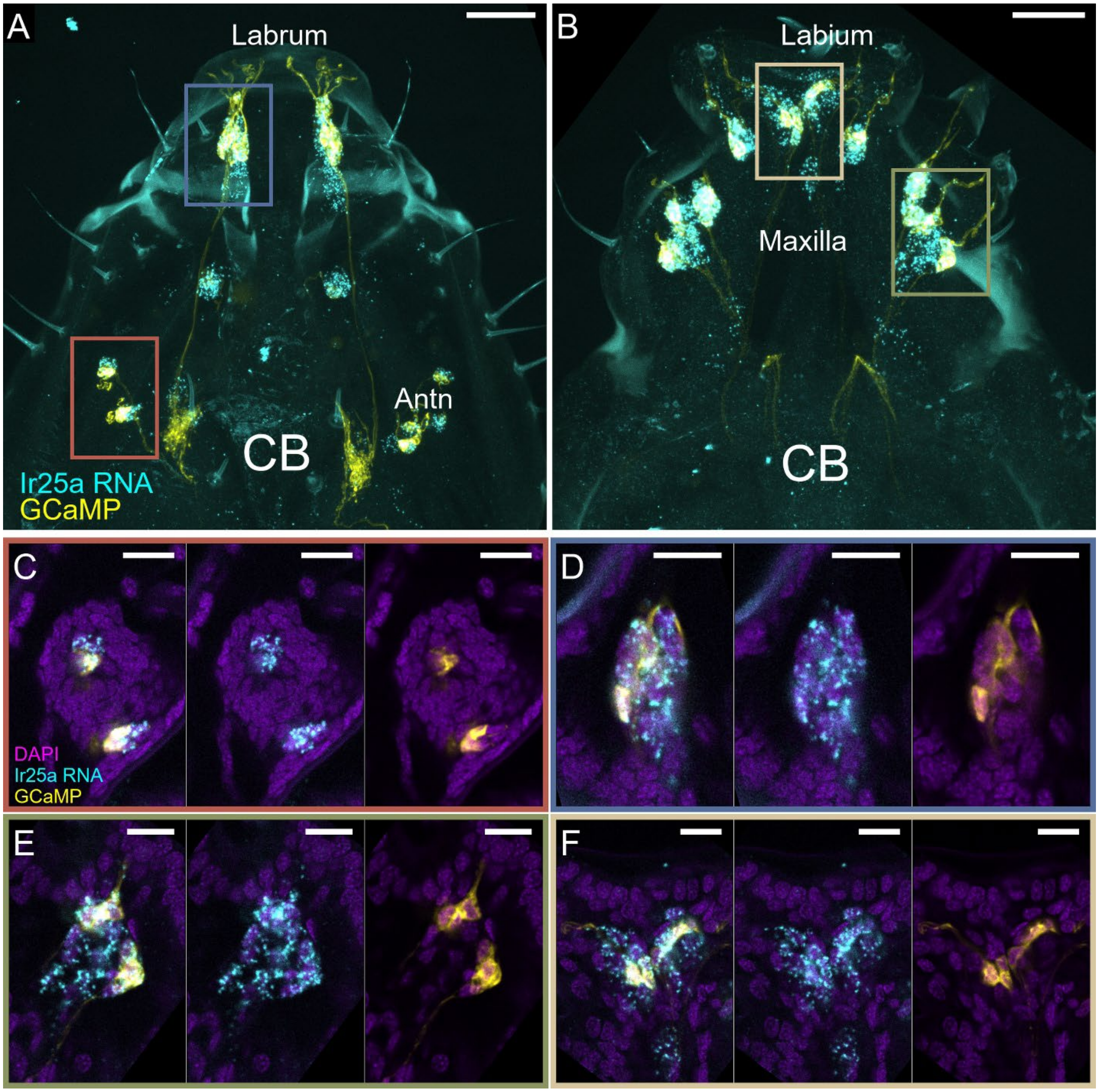

Fig. S7. Transgenic GCaMP expression under control of the *Ir25a* promotor fragment recapitulates endogenous *Ir25a* expression in fourth-instar larvae. (A and B) Maximum-intensity projections of the upper (A) and lower (B) portions of the larval head showing native GCaMP fluorescence, without antibody amplification, in RNA-FISH positive cells for endogenous *Ir25a*. The antennal and labral regions are shown in (A), and the maxillary and labial regions are shown in (B). Antn, antenna; CB, central brain. (C–F) Individual z-slices showing overlap between GCaMP fluorescence and endogenous *Ir25a* RNA-FISH signal in the antennal (C), labral (D), maxillary (E), and labial (F) regions. DAPI is shown in magenta, *Ir25a* RNA-FISH signal in cyan, and GCaMP fluorescence in yellow. Border colors correspond to the anatomical regions defined in Fig. 1B. Scale bars: 100 µm (A and B) and 10 µm (C–F). Images are representative of one fourth-instar larva.

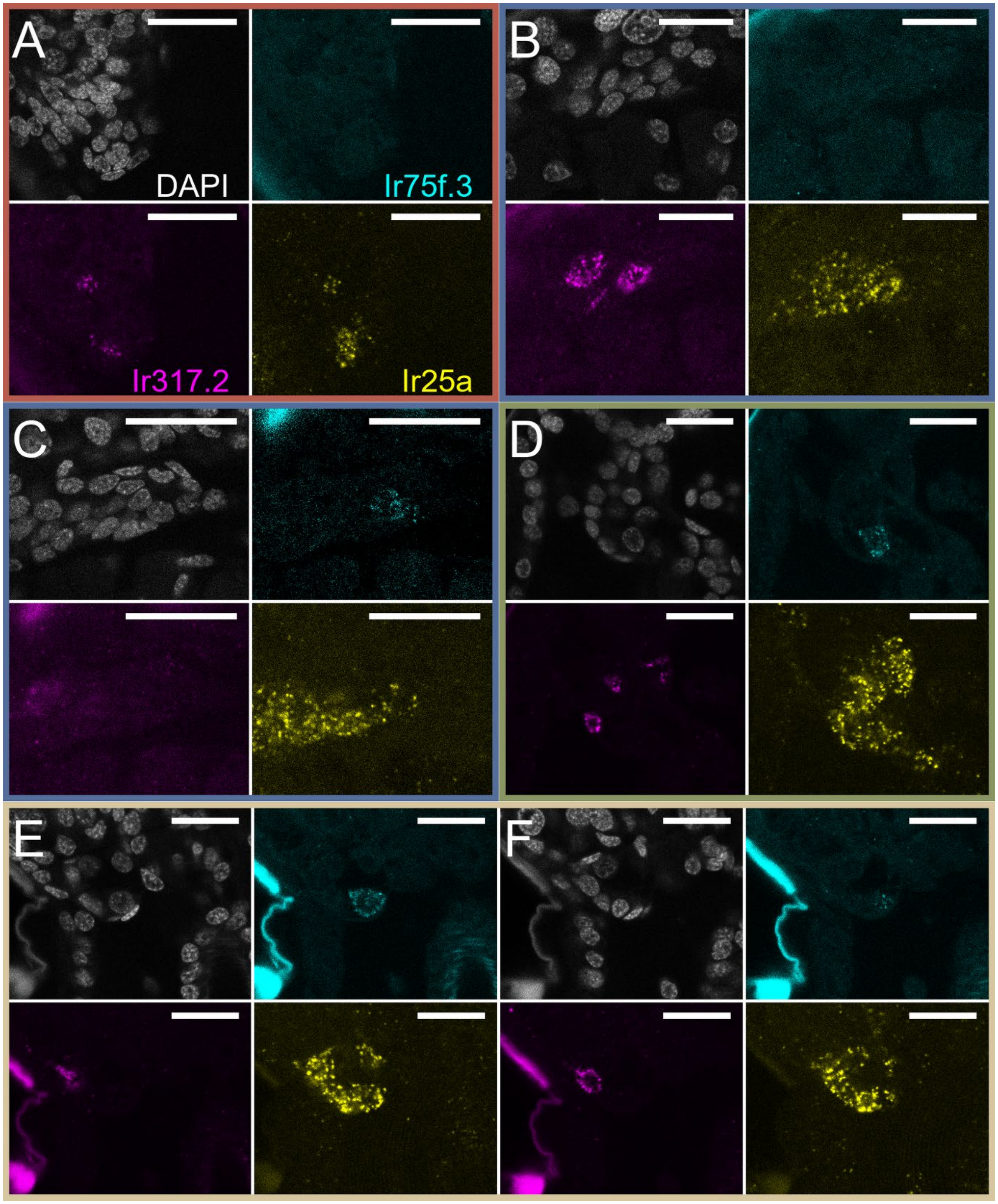

Fig. S8. Regional expression of *Ir75f.3* and *Ir317.2* in *Ir25a*-positive larval sensory neurons. (A–F) Representative individual z-slices showing separate RNA-FISH channels for *Ir75f.3* (cyan), *Ir317.2* (magenta), and *Ir25a* (yellow) in the antennal region (A), labral region (B and C), maxillary region (D), and labial region (E and F). DAPI is shown in white. Border colors correspond to the anatomical regions defined in Fig. 1B. Scale bars: 15 µm (A–D and F) and 10 µm (E).

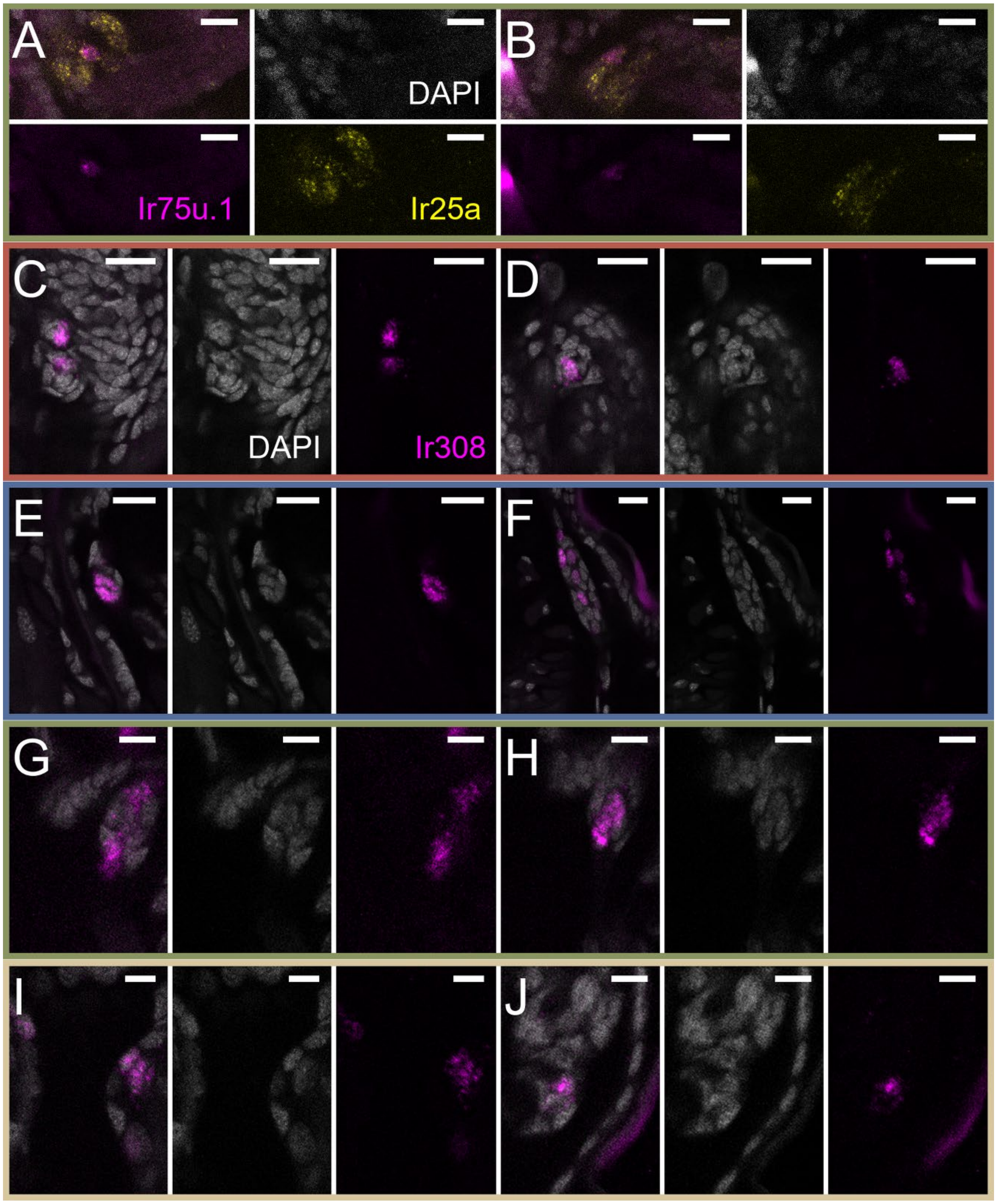

Fig. S9. Restricted *Ir75u.1* and broad *Ir308* expression in larval head sensory organs**.** (A and B) Composite and individual RNA-FISH channels showing *Ir75u.1* (magenta) and *Ir25a* (yellow) expression in the maxillary region. *Ir75u.1* was detected in a single *Ir25a*-positive cell on each maxilla. (C–J) Composite and individual channels showing *Ir308* RNA-FISH signal (magenta) in the antennal (C and D), labral (E and F), maxillary (G and H), and labial (I and J) regions. DAPI is shown in white. Border colors correspond to the anatomical regions defined in Fig. 1B. Scale bars: 10 μm (A–F) and 5 μm (G–J).

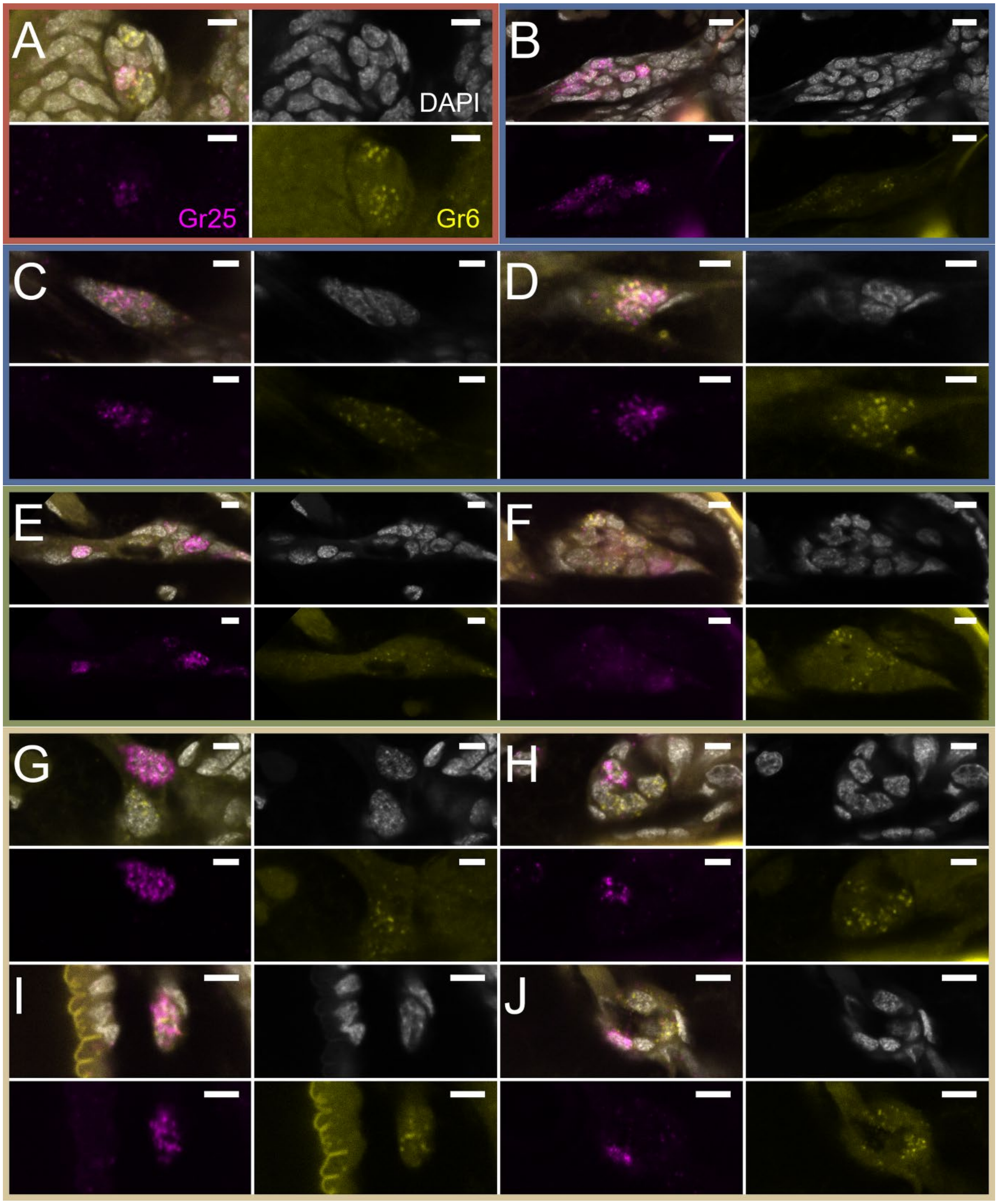

Fig. S10. Regional expression of *Gr25* and *Gr6* in larval head sensory organs. (A–J) Representative composite and individual RNA-FISH channels showing *Gr25* (magenta) and *Gr6* (yellow) expression in the antennal (A), labral (B–D), maxillary (E and F), and labial (G–J) regions. DAPI is shown in white. Border colors correspond to the anatomical regions defined in Fig. 1B. Scale bars: 5 µm.

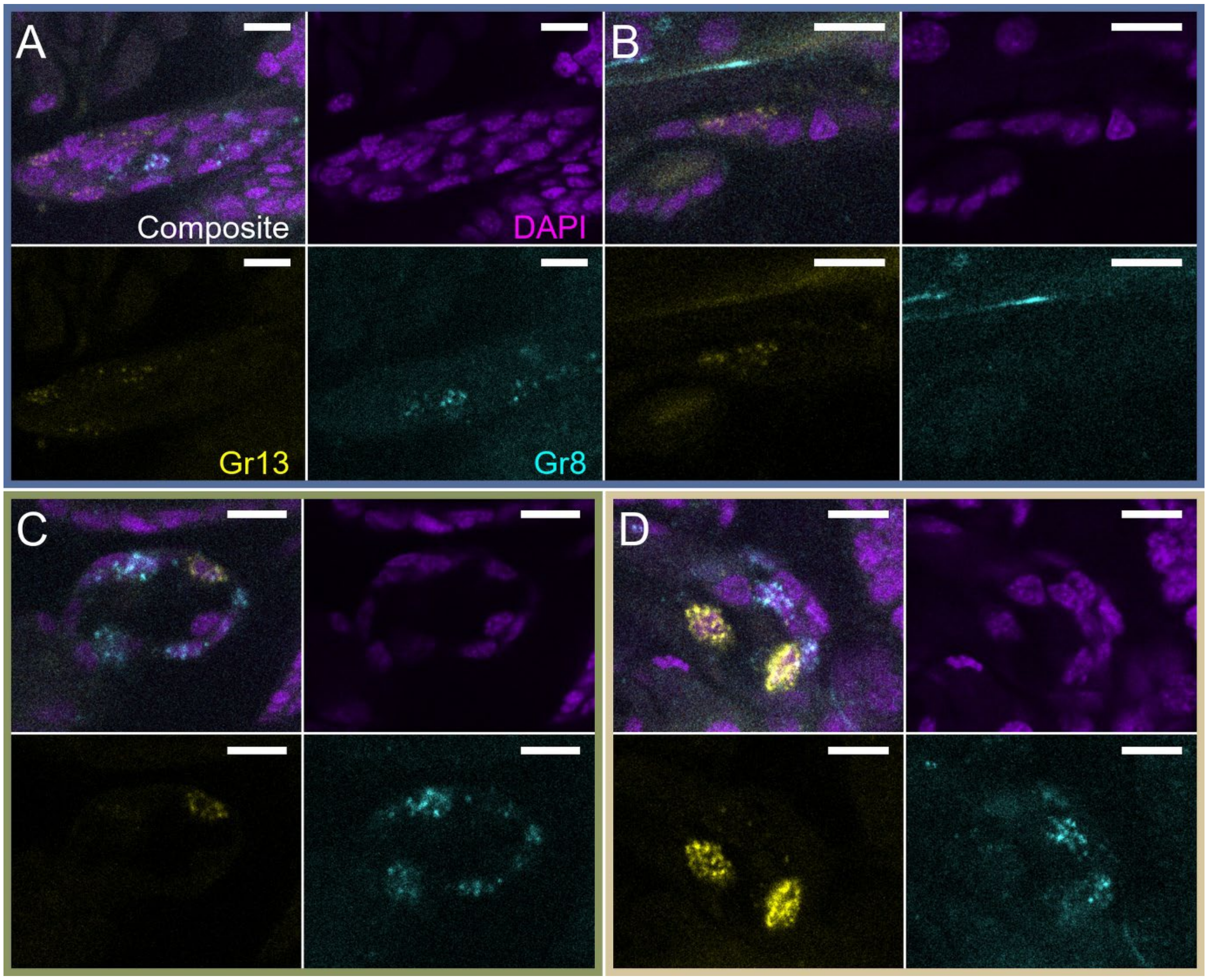

Fig. S11. Regional expression of *Gr13* and *Gr8* in larval head sensory organs. (A–D) Representative composite and individual RNA-FISH channels showing *Gr13* (yellow) and *Gr8* (cyan) expression in the labral (A and B), maxillary (C), and labial (D) regions. DAPI is shown in magenta. Border colors correspond to the anatomical regions defined in Fig. 1B. Scale bars: 15 µm.

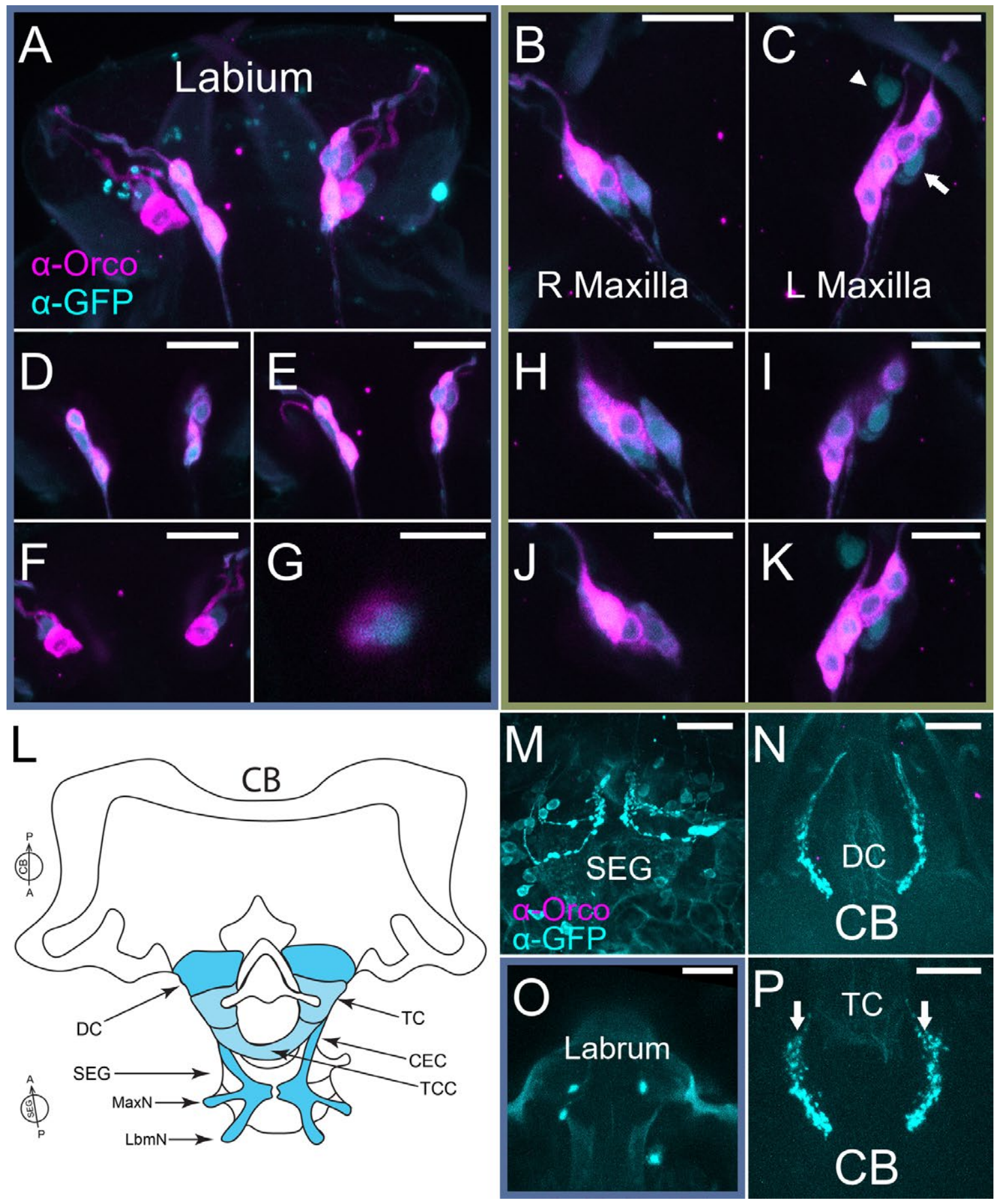

Fig. S12. Transgenic GCaMP expression under control of an *Orco* promotor fragment largely recapitulates endogenous *Orco* expression in fourth-instar larval chemosensory neurons. (A–K) Immunostaining for Orco (magenta) and GCaMP, detected using an anti-GFP antibody (cyan), in a fourth-instar *Orco*>GCaMP larva. GCaMP signal overlaps with Orco-positive sensory neurons in the labium (A and D–G) and maxilla (B, C, and H–K). In (C), the arrowhead marks a GCaMP-positive cell without detectable Orco staining, whereas the arrow marks a GCaMP-positive cell with faint Orco staining. Panels (D–G) and (H–K) are serial optical sections arranged from dorsal to ventral, revealing distinct groups of Orco-positive neurons. (L) Schematic of the central regions containing GCaMP signal in the *Orco*>GCaMP line, corresponding to panels (M–P). For clarity, the central brain (CB) and subesophageal ganglion (SEG) are shown perpendicular to one another, although they lie approximately parallel within the head. Compass symbols indicate their anatomical orientations in situ. (M and N) GCaMP-positive projections from the maxillary and labial nerves into the SEG (M) and deutocerebral region (DC) (N). GCaMP is present in the axons of Orco-positive sensory neurons, whereas Orco protein is not detectably localized to these projections. (O and P) A small population of GCaMP-positive, Orco-negative labral cells (O) projects to a weakly labeled tritocerebral region (TC) dorsal and anterior to the deutocerebrum (arrows in (P)). CEC, circumesophageal connective; TCC, tritocerebral commissure. R and L indicate the larva's anatomical right and left, respectively. Border colors correspond to the anatomical regions defined in Fig. 1B. Scale bars: 20 µm (A–F); 10 µm (G); 15 µm (H–K) and (M–P).

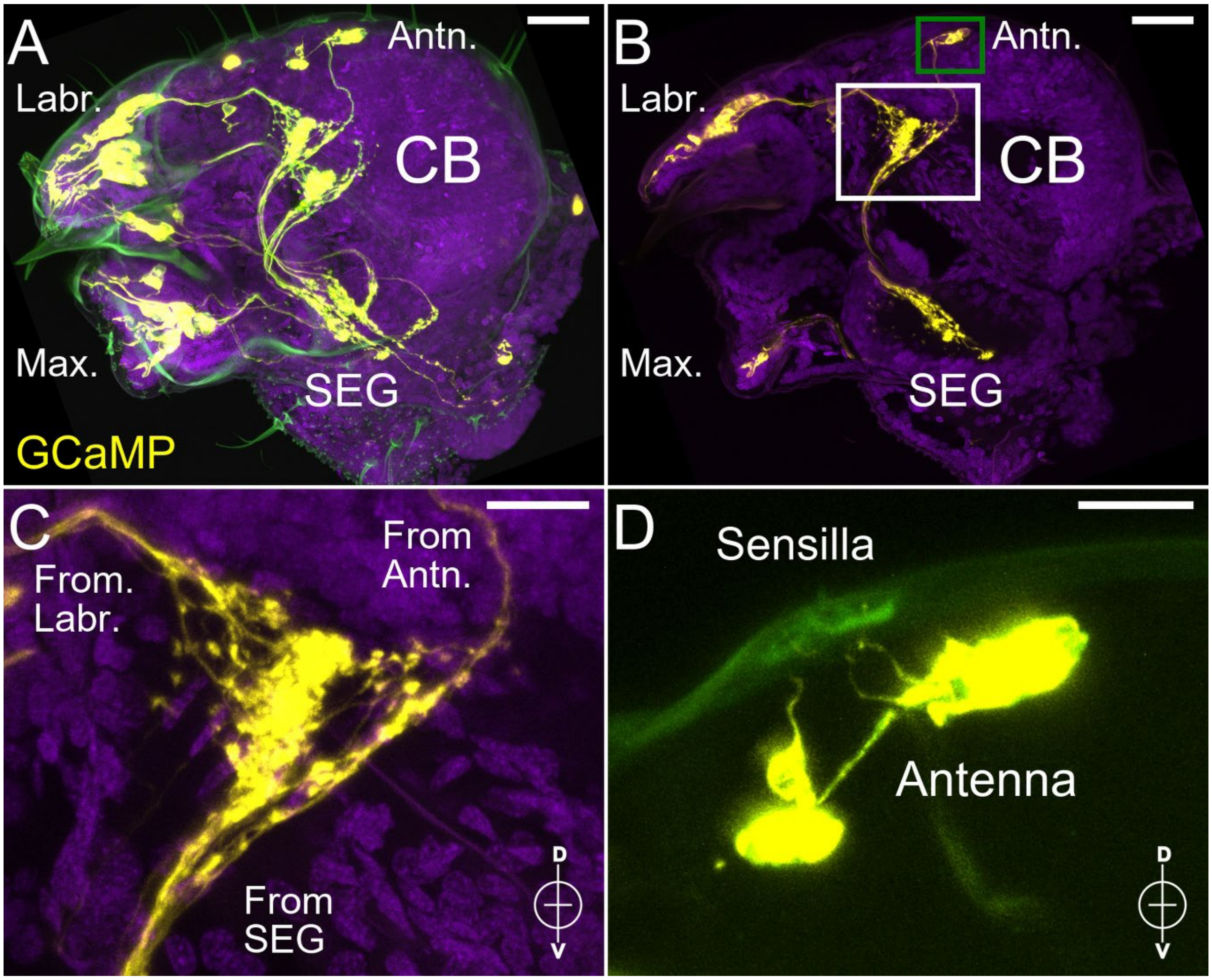

Fig. S13. *Ir25a*>GCaMP–positive sensory projections from multiple larval head regions. (A) Maximum-intensity projection (MIP) of a fourth-instar *Ir25a*>GCaMP larval head showing GCaMP (yellow), tdTomato (green), and DAPI (magenta). (B) Partial MIP of a subset of optical sections from (A) highlighting projections from multiple sensory regions into the central brain (white box) and the antennal neurons and sensilla (green box). GCaMP is shown in yellow and DAPI in magenta. (C) Magnification of the white-boxed region in (B) showing projections entering the central brain from the SEG, labrum, and antenna. The labeled projections shown from each region originate from one side of the head. (D) Magnification of the green-boxed region in (B) showing antennal neurons on one side of the head innervating the three antennal papilla sensilla. CB, central brain; SEG, subesophageal ganglion; Labr., labrum; Max., maxilla; Antn., antenna. Orientation markers indicate dorsal (D), ventral (V), anterior (left), and posterior (right). Scale bars: 25 µm (A and B); 10 µm (C and D).

Table S1. Overview of chemosensory receptor-expressing cells in the clonal raider ant larval head.

Gene	Antennae	Dorsal esophagus	Labrum	Maxillae	Labium	Total
Orco	0	0	0	11	9	20
OrW1	0	0	0	2	0	2
OrE3	0	0	0	0	2	2
Ir25a	22	18	64	82	52	238
Ir8a	20	18	86	63	45	232
Ir76b	6	4	34	29	24	96
Ir317.2	6	0	7	7	4	24
Ir75f.3	0	0	5	3	2	11
Ir308	6	8	33	16	14	77
Ir75u.1	0	0	0	2	0	2
Gr25	6	4	27	19	15	71
Gr6	8	4	40	21	16	89
Gr8	2	0	25	39	30	96
Gr13	11	5	20	8	10	54

Values are manual counts of receptor-expressing cells detected by RNA-FISH or, for Orco, IHC, with all samples imaged with confocal microscopy. Except for Ir8a, counts were averaged across three biological replicates and rounded to the nearest whole cell; Ir8a counts are from one biological sample. Totals were calculated from replicate-level whole-head counts before rounding; therefore, the sum of the rounded regional values may differ from the reported total. *Ir25a* counts are also reported in Table S3B.

Table S2. Percentage of reference gene-positive cells coexpressing the indicated gene by anatomical region and across the whole head.

A. By anatomical region

Reference gene	Coexpressed gene(s)	Antennae	Dorsal esophagus	Labrum	Maxillae	Labium
Ir8a	*Ir25a*	100	94	95	97	98
Ir76b	*Ir25a*	96	83	84	89	88
Gr25	*Ir25a*	100	50	80	39	77
Gr25	*Ir25a, Orco*	0	8	0	6	11

B. Whole head

Reference gene	Coexpressed gene(s)	# Reference gene-positive cells	# Co-labeled cells	Whole-head coexpression (%)
Ir8a	*Ir25a*	233	225	97
Ir76b	*Ir25a*	294	257	87
Gr25	*Ir25a*	109	67	61
Gr25	*Ir25a, Orco*	109	6	5

Values indicate the percentage of reference gene-positive cells that also expressed the indicated coexpressed gene(s), based on manual counts from RNA-FISH images acquired by confocal microscopy. When multiple genes are listed, values indicate cells positive for all indicated genes. In (A), *Ir76b*/*Ir25a* percentages were averaged across three biological replicates; all other comparisons are from one biological sample each. In (B), the percentage of cells with whole-head coexpression was calculated from cell counts pooled across anatomical regions and, for *Ir76b*/*Ir25a*, biological replicates. All percentages were rounded to the nearest whole percent.

Table S3. Validation of *Orco*-GCaMP and *Ir25a*-GCaMP transgenic lines.

A. *Orco*-GCaMP

Region	Total Orco+ cells	GCaMP+ only	Co-labeled	GCaMP concordance (%)	Orco coverage (%)
Antennae	0	0	0	—	—
Dorsal esophagus	0	0	0	—	—
Labrum	0	4	0	0	—
Maxillae	10	1	10	91	100
Labium	11	0	10	100	91
**Total**	**21**	**5**	**20**	**80**	**95**

B. *Ir25a*-GCaMP

Region	Total *Ir25a*+ cells	GCaMP+ only	Co-labeled	GCaMP concordance (%)	*Ir25a* coverage (%)
Antennae	22	0	13	100	59
Dorsal esophagus	18	0	2	100	11
Labrum	64	0	30	100	47
Maxillae	82	0	35	100	42
Labium	52	0	15	100	30
**Total**	**239**	**0**	**95**	**100**	**40**

Endogenous Orco and *Ir25a* were detected by immunohistochemistry and RNA-FISH, respectively. Concordance is the percentage of GCaMP+ cells positive for the corresponding endogenous marker; coverage is the percentage of endogenous-marker+ cells captured by GCaMP. Orco-GCaMP counts are from one biological sample. For *Ir25a*-GCaMP, cell counts are means of three biological replicates, rounded to the nearest whole cell, whereas concordance and coverage were calculated for each biological replicate and then averaged.
